# Supplementary material for: Two Faces of CwlM, an Essential PknB Substrate, in Mycobacterium tuberculosis
Source: Cell Rep. 2018 Oct 2;25(1):57–67.e5. doi: 10.1016/j.celrep.2018.09.004 (PMC6180346; doi:10.1016/j.celrep.2018.09.004)
Supplement: Document S1. Figures S1–S7 and Tables S2–S5 [file mmc1.pdf]

Cell Reports, Volume *25*

Supplemental Information

Two Faces of CwlM, an Essential PknB Substrate, in *Mycobacterium tuberculosis*

Obolbek Turapov, Francesca Forti, Baleegh Kadhim, Daniela Ghisotti, Jad Sassine, Anna Straatman-Iwanowska, Andrew R. Bottrill, Patrick J. Moynihan, Russell Wallis, Philippe Barthe, Martin Cohen-Gonsaud, Paul Ajuh, Waldemar Vollmer, and Galina V. Mukamolova

SUPPLEMENTAL FIGURES

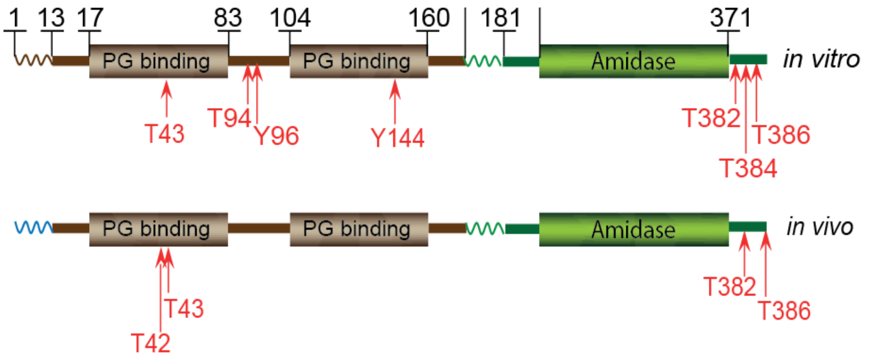

B

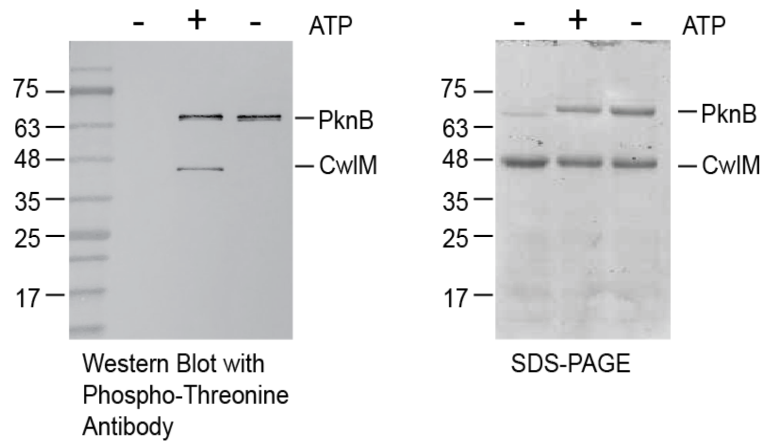

C

Peptide	Phospho residue	Observed mass (charge)	Actual mass
AALTALGMLDHQEEDLTTGR	T43	1111.52 (2)	2221.03
RLGARTLYHQFGAPLYGDDVATLQAR	T94	938.47 (3)	2814.4
RLGARTLYHQFGAPLYGDDVATLQAR	Y96	704.11 (4)	2812.4
EYGLAADGICGPETLR	Y144	595.93 (3)	1784.78
NDRPTGTFTFAELLAHELSVER	T382	862.08 (3)<br>646.82 (4)	2583.22<br>2583.22
NDRPTGTFTFAELLAHELSVER	T384	646.81 (4)<br>862.09 (4)	2583.22
NDRPTGTFTFAELLAHELSVER	T382<br>T384	888.74 (3)	2663.2
NDRPTGTFTFAELLAHELSVER	T382<br>T386	888.41 (3)	2662.2
NDRPTGTFTFAELLAHELSVER	T386	861.76 (3)	2582.26
NDRPTGTFTFAELLAHELSVER	T384<br>T386	888.41 (3)	2662.20

Figure S1. Phosphorylation of CwlM by PknB *in vitro*. Related to Table 1. (A) Schematic representation of phosphosites detected *in vitro* (top) and in the phosphoproteomics study (bottom). (B) A recombinant CwlM was phosphorylated by the recombinant enzymatic domain of PknB. Detection of phosphorylated proteins by Western blot analysis using phospho-threoinine antibody. (C) Phosphopeptides detected by mass-spectrometry.

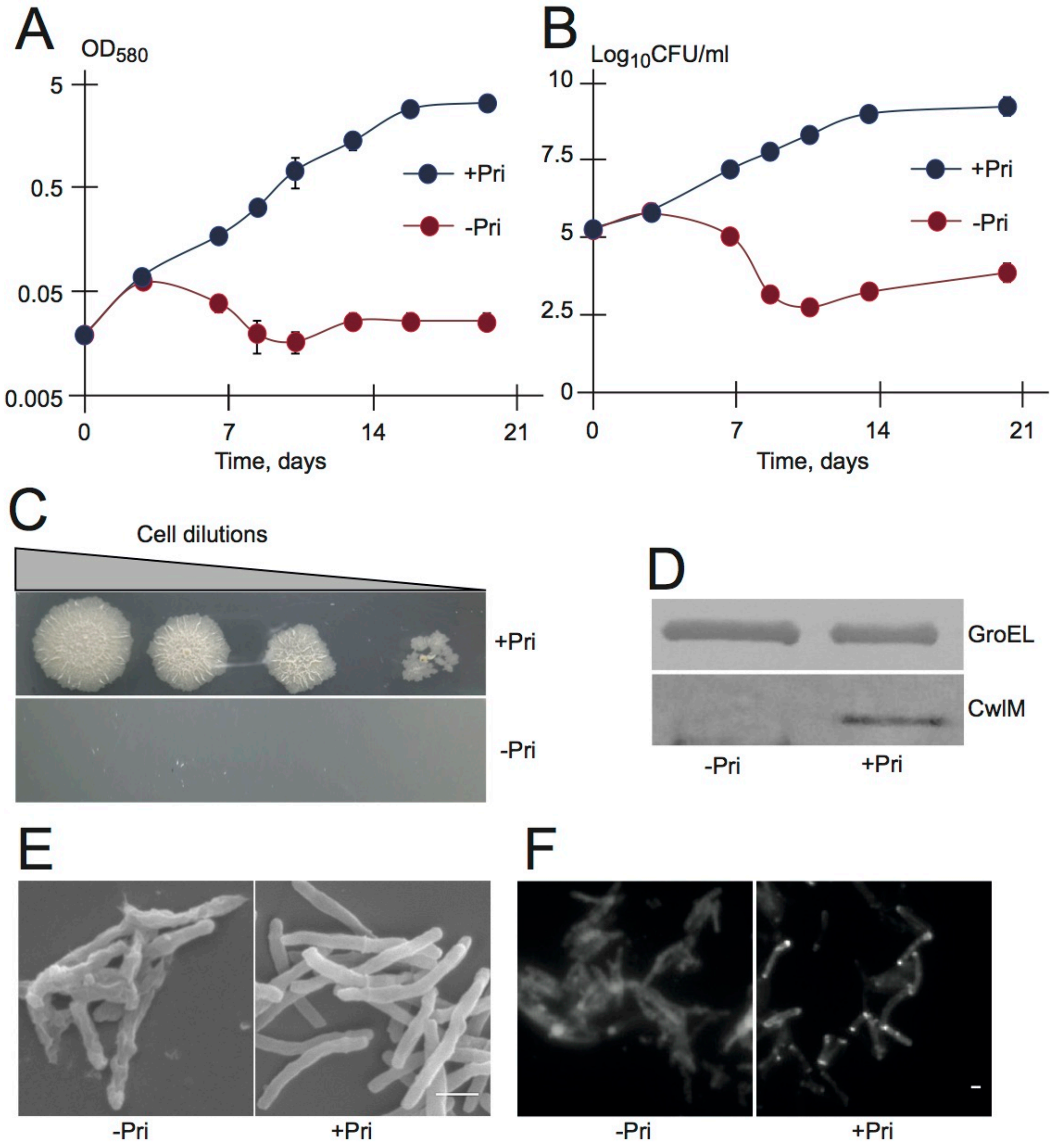

Figure S2. CwlM is essential for growth of *M. tuberculosis.* Related to Figure 2. See also Figure S3 and Table S3. A conditional *cwlM* mutant was grown in 7H9 medium with and without pristinamycin (Pri) at 37°C with shaking. Growth was monitored by measurement of OD at 580 nm (A) or by CFU counting on 7H10 agar (B). Data are represented as mean±SEM. (C) Growth of *cwlM*-CM on agar. (D) Detection of CwlM using western blot with anti-CwlM antibody. (E) Scanning electron micrographs of CwlM-producing and CwlM-depleted mycobacteria grown in standard 7H9 medium. (F) Detection of nascent peptidoglycan by Van-BODIPY labelling, data shown for mycobacteria grown in 7H9 medium, similar results were obtained in SMM (not shown for clarity). Scale bars – 1 μm.

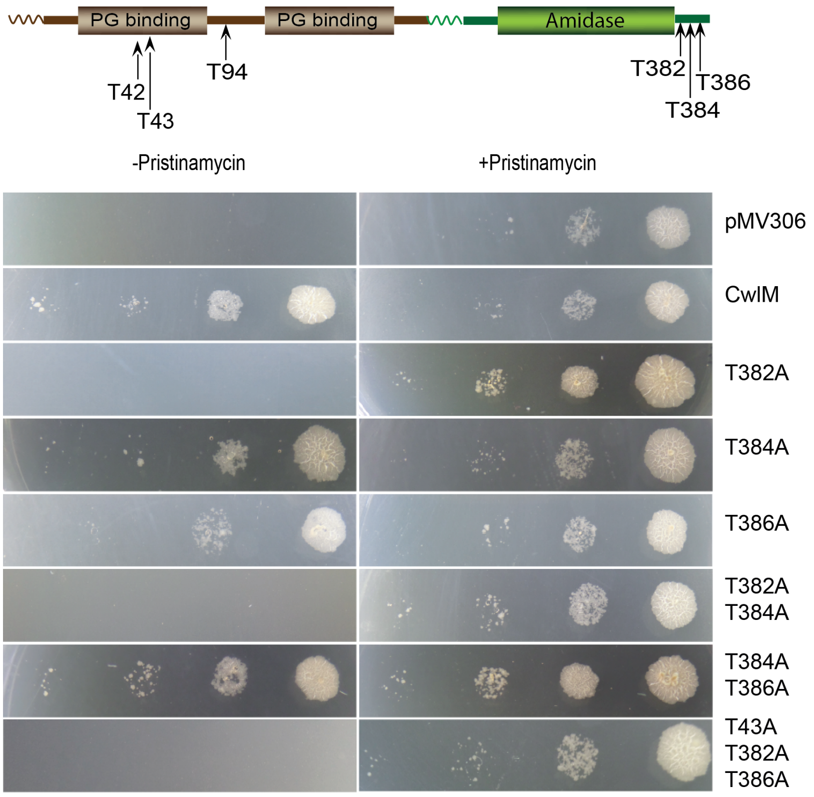

A

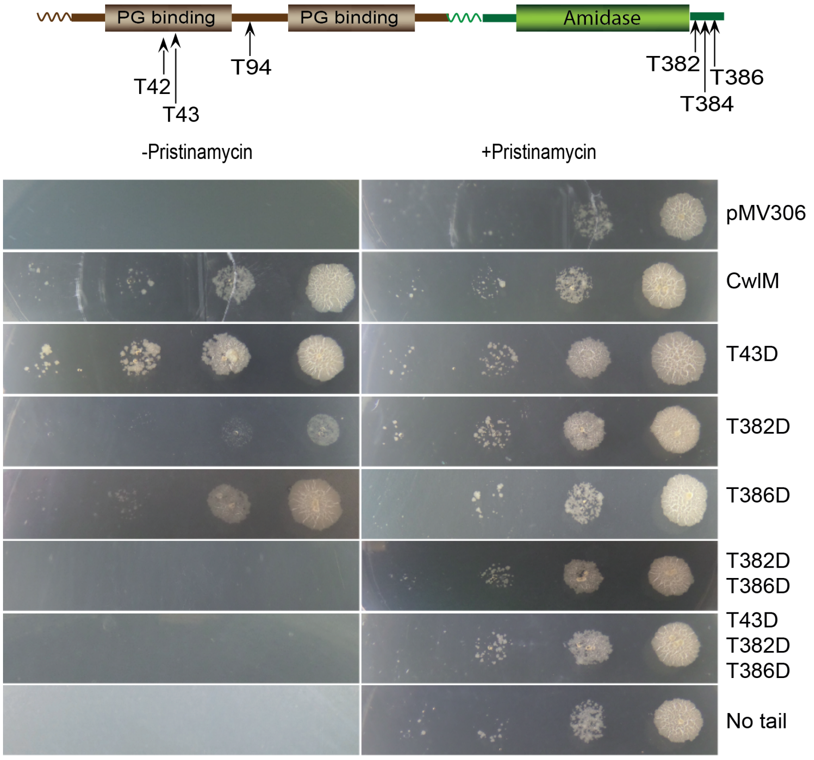

B

Figure S3. Growth of phosphoablative (A) and phosphomimetic (B) *M. tuberculosis* CwlM mutants on 7H10 agar. Related to Figure 2. See also Table S3. Serially diluted mycobacteria were plated on 7H10 agar with kanamycin and hygromycin +/-pristinamycin and incubated for 4 weeks at 37°C.

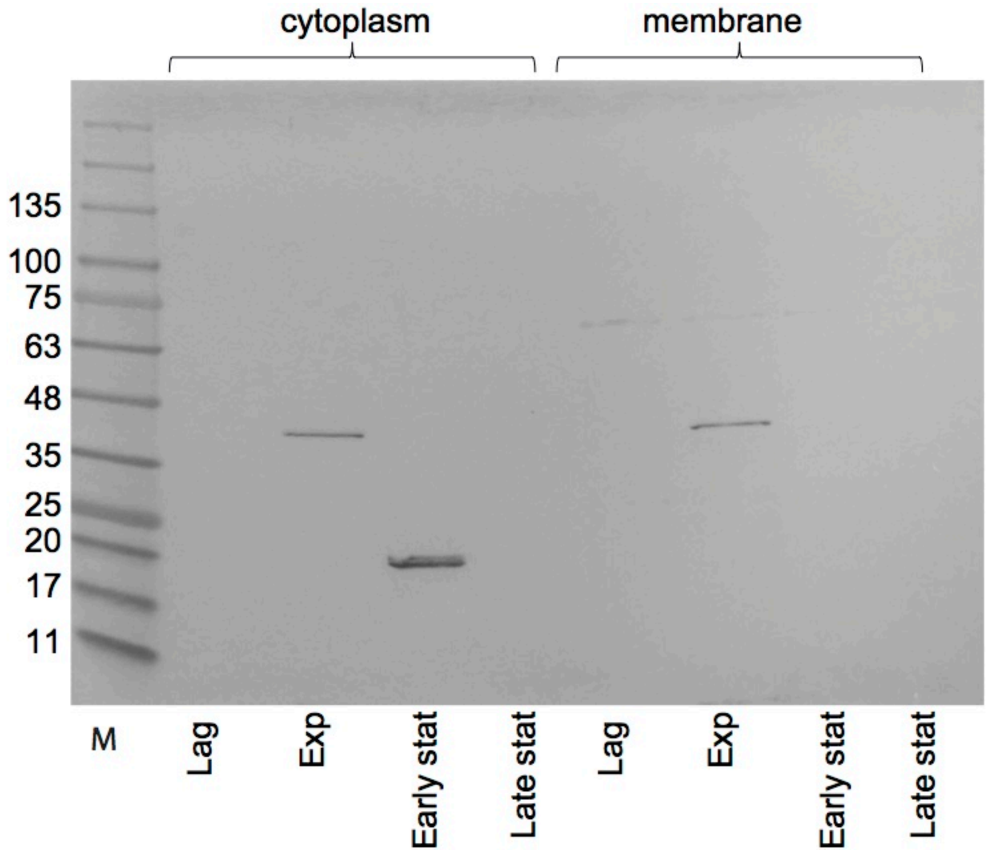

Figure S4. Detection of CwlM in cytoplasm and membrane fractions of *M. smegmatis* during different growth stages*.* Related to Figure 3. Bacteria were grown inoculated in 7H9 medium and incubated for 1 hour (lag-phase, Lag), grown to OD 0.8 (exponential phase, Exp), 3.6 (early stationary phase, Early stat) and 7.4 (late stationary phase, Late stat) at 37°C with shaking. Fractionation and Western blot analysis using the anti-CwlM antibody were done as described in Methods.

-TRIMETHOPRIM

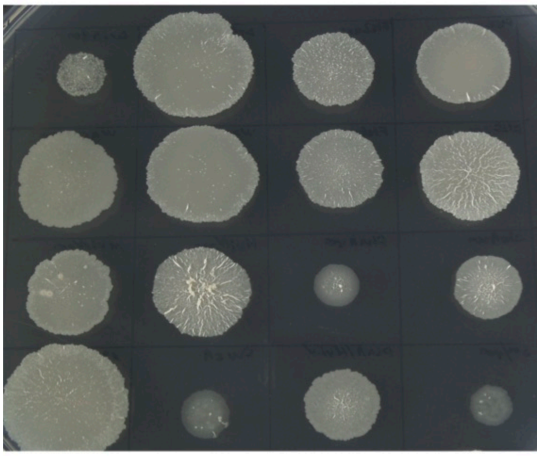

+TRIMETHOPRIM

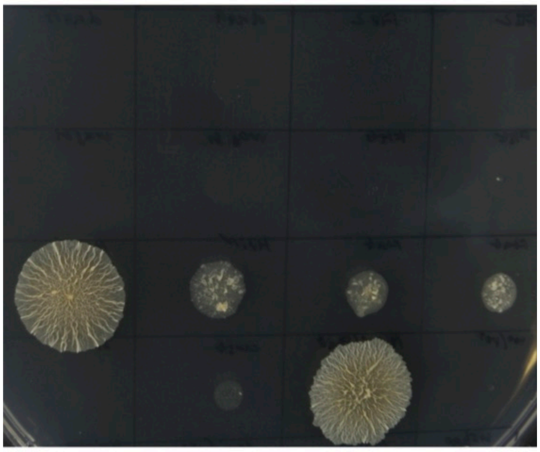

MAP

DnaA$_{400}$<br>CwlM$_{300}$	DnaA$_{200}$<br>CwlM$_{100}$	FtsE$_{400}$<br>CwlM$_{300}$	FtsE$_{200}$<br>CwlM$_{100}$
Wag31$_{400}$<br>CwlM$_{300}$	Wag31$_{200}$<br>CwlM$_{100}$	FtsZ$_{400}$<br>CwlM$_{300}$	FtsZ$_{200}$<br>CwlM$_{100}$
MurJ$_{icd400}$<br>CwlM$_{300}$	MurJ$_{icd200}$<br>CwlM$_{100}$	FhaA$_{400}$<br>CwlM$_{300}$	FhaA$_{200}$<br>CwlM$_{100}$
pUAB200<br>CwlM$_{100}$	CwsA$_{400}$<br>CwlM$_{300}$	MurJ$_{icd100}$<br>FhaA$_{200}$	pUAB300<br>pUAB400

Figure S5. Detection interactions of CwlM with other proteins by a mycobacterial protein fragment complementation assay. Related to Figure 5. See also Table S4. 300/400 is a negative empty plasmid control (pUAB300+pUAB400); CwlM/400 – control (pUAB300::*cwlM*+pUAB400); MurJ$_{ICD}$/FhaA – positive control (pUAB100:: *murJN$_{icd}$* +pUAB200::*fhaA*). Growth in the presence of trimethoprim indicates interactions between proteins fused to dihydrofolate-reductase domains. MAP shows the position of strains used in the assays (strains are detailed in Table S2).

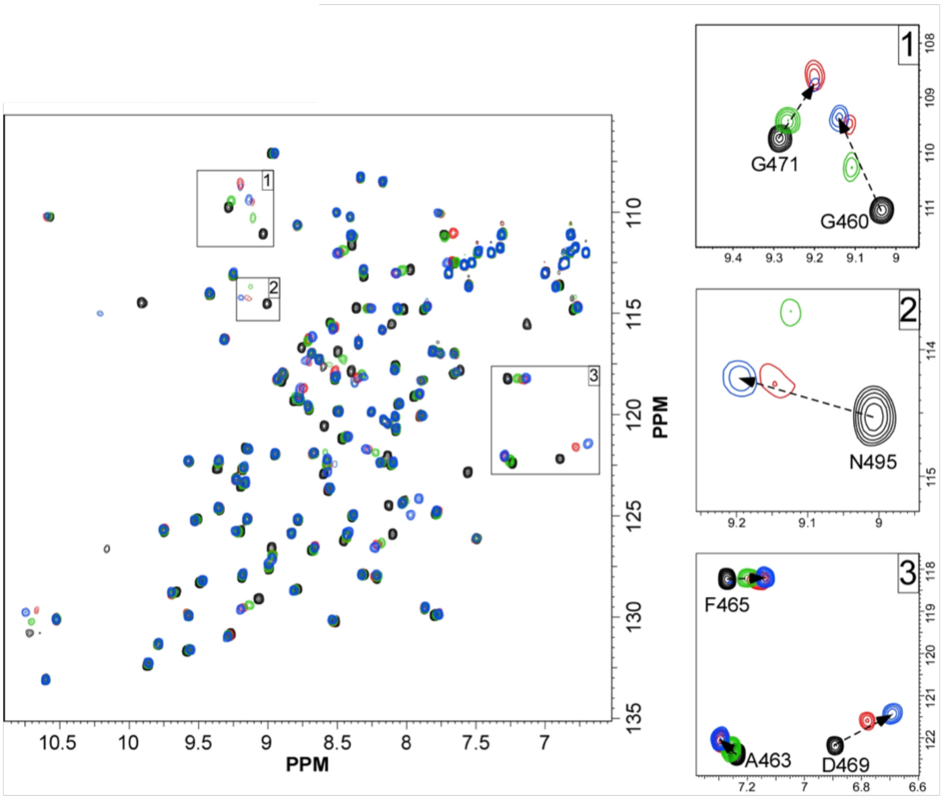

Figure S6. Superimposition of [^{1}H-^{15}N] HSQC spectra of Rv0020c FHA domain alone and in presence of 3 different phospho-peptides. Related to Figure 5. Superimpositions of [^{1}H-^{15}N] HSQC spectra of uniformly labelled samples Rv0020c FHA domain (50 µM) alone (black) and in presence of 50 µM of T382 phospho-peptide (red) or T386 phospho-peptide (green) or the double T382 and T386 phospho-peptide (blue). Three areas of the HSQC spectrum are expanded to the right. In each zoom, peaks are assigned and an arrow indicates the displacement of the peak when titrating with the double T382 and T386 phospho-peptide. The displacement observed for the double phosphor-peptide corresponds to the displacement observed for the T382 phospho-peptide.

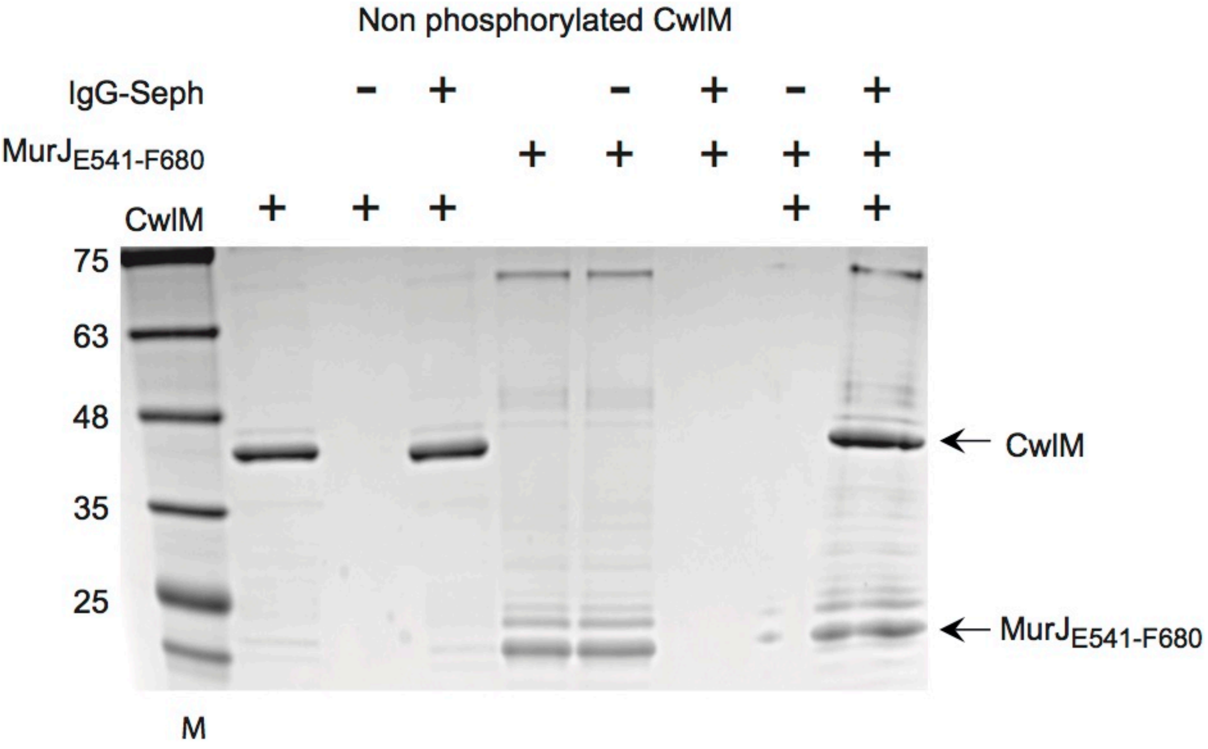

Figure S7. CwlM interacts with an E541-F680 linker of $MurJ_{ICD}$. Related to Figure 4. Recombinant proteins were immunoprecipitated and analysed by SDS-PAGE. M- protein markers; "+" – reagent added; "-" designates flow-through fractions.

SUPPLEMENTAL TABLES

Table S2. Strains generated in the study. Related to Figure 1-4.

Strain name	Strain description	Plasmid	Comments
***Mycobacterium tuberculosis* H37Rv strains**			
pknB-CM	PknB conditional mutant	pAZI9479::*pkn*	PknB depletion
cwlM-CM	CwlM conditional mutant	pAZI9479::*cwlM*	CwlM depletion
cwlM-CM$_{pMV}$	Empty plasmid control	pMV306	*cwlM*-CM complementation
cwlM-CM$_{WT}$	CM-CwlM wild type *cwlM* complemented	pMV306::*cwlM*	*cwlM*-CM complementation
cwlM-CM$_{T42A}$	T42A CwlM variant	pMV306::*cwlMT42A*	*cwlM*-CM complementation
cwlM-CM$_{T43A}$	T43A CwlM variant	pMV306::*cwlMT43A*	*cwlM*-CM complementation
cwlM-CM $_{T42A+T43A}$	T42A+T43A CwlM variant	pMV306::*cwlMT42A +T43A*	*cwlM*-CM complementation
cwlM-CM $_{T94A}$	T94A CwlM variant	pMV306::*cwlMT94A*	*cwlM*-CM complementation
cwlM-CM $_{T382A}$	T382A CwlM variant	pMV306::*cwlMT382 A*	*cwlM*-CM complementation
cwlM-CM $_{T384A}$	T384A CwlM variant	pMV306::*cwlMT384 A*	*cwlM*-CM complementation
cwlM-CM $_{T386A}$	T386A CwlM variant	pMV306::*cwlMT386 A*	*cwlM*-CM complementation
cwlM-CM $_{T382A+T384A}$	T382A +T384A CwlM variant	pMV306::*cwlMT382 A+384A*	*cwlM*-CM complementation
cwlM-CM $_{T382A+T384A+T386A}$	T382A +T384A+T386A CwlM variant	pMV306::*cwlMT382 A+384A+T386A*	*cwlM*-CM complementation
cwlM-CM $_{T43A+T382A+T386A}$	T43A+T382A +T384A CwlM variant	pMV306::*cwlMT43A +T382A+386A*	*cwlM*-CM complementation
cwlM-CM $_{T384A+T386A}$	T384A +T386A CwlM variant	pMV306::*cwlMT384 A+T386A*	*cwlM*-CM complementation
cwlM-CM$_{T43D}$	T43D CwlM variant	pMV306::*cwlMT43D*	*cwlM*-CM complementation
cwlM-CM$_{T382D}$	T382D CwlM variant	pMV306::*cwlMT382 D*	*cwlM*-CM complementation
cwlM-CM$_{T386D}$	T386D CwlM variant	pMV306::*cwlMT386 D*	*cwlM*-CM complementation
cwlM-CM $_{T382D+T386D}$	T382D+T386D CwlM variant	pMV306::*cwlMT382 D+T386D*	*cwlM*-CM complementation
cwlM-CM $_{T43D+T382D +T386D}$	T43D+T382D+T386D CwlM variant	pMV306::*cwlMT43D +T382D+T386D*	*cwlM*-CM complementation
cwlM-CM$_{R2}$	Truncated CwlM version (M1- K370)	pMV306::*cwlMR2*	*cwlM*-CM complementation
***Mycobacterium smegmatis* mc^{2}155 strains**			
pUAB300+ pUAB400	Empty plasmid control	pUAB300 pUAB400	Mycobacterial protein fragment complementation (M-PFC)

$CwlM_{100}$/ $DnaA_{200}$	Interaction strain	pUAB100::*cwl* pUAB200::*dnaA*	M-PFC
$CwlM_{300}$/ $DnaA_{400}$	Interaction strain	pUAB300::*cwlM* pUAB400::*dnaA*	M-PFC
$CwlM_{100}$/ $FtsE_{200}$	Interaction strain	pUAB100::*cwlM* pUAB200::*ftsEA*	M-PFC
$CwlM_{300}$/ $FtsE_{400}$	Interaction strain	pUAB300::*cwlM* pUAB400::*ftsE*	M-PFC
$CwlM_{100}$/ $FtsZ_{200}$	Interaction strain	pUAB100::*cwlM* pUAB200::*ftsZ*	M-PFC
$CwlM_{300}$/ $FtsZ_{400}$	Interaction strain	pUAB300::*cwlM* pUAB400::*ftsZ*	M-PFC
$CwlM_{100}$/ $Wag31_{200}$	Interaction strain	pUAB100::*cwlM* pUAB200::*wag31*	M-PFC
$CwlM_{300}$/ $Wag31_{400}$	Interaction strain	pUAB300::*cwlM* pUAB400::*wag31*	M-PFC
$CwlM_{100}$/ $FhaA_{200}$	Interaction strain	pUAB100::*cwlM* pUAB200::*fhaA*	M-PFC
$CwlM_{300}$/ $FhaA_{400}$	Interaction strain	pUAB300::*cwlM* pUAB400::*fhaA*	M-PFC
$CwlM_{100}$/ $MurJ_{icd200}$	Interaction strain	pUAB100::*cwlM* pUAB200::$murJ_{icd}$	M-PFC
$CwlM_{300}$/ $MurJ_{icd400}$	Interaction strain	pUAB300::*cwlM* pUAB400::$murJ_{icd}$	M-PFC
$CwlM_{300}$/ $CwsA_{400}$	Interaction strain	pUAB300::*cwlM* pUAB400::*cwsA*	M-PFC
$MurJ_{icd100}$/ $FhaA_{200}$	Interaction strain	pUAB100:: $murJ_{icd}$ pUAB200::*fhaA*	M-PFC
$CwlM_{100}$/200	Interaction strain	pUAB100::*cwlM* pUAB200	M-PFC
$CwlM_{300}$/ 400	Interaction strain	pUAB300::*cwlM* pUAB400	M-PFC
$MurJ_{icd200}$/ 100	Interaction strain	pUAB100 pUAB200::$murJ_{icd}$	M-PFC
$MurJ_{icd400}$/300	Interaction strain	pUAB300 pUAB400::$murJ_{icd}$	M-PFC
C41(DE3) pET CwlM	6xHis-CwlM expression strain	pET15bTEV::*cwlM*	M-PFC
C41 (DE3) pET $MurJ_{icd}$	6xHis-$MurJ_{icd}$ expression strain	pET15bTEV::$murJ_{icd}$	Recombinant $MurJ_{icd}$
C41 (DE3)pET $MurJ_{E541-F680}$	6xHis-$MurJ_{E541-F680}$ expression strain	pET15bTEV::$murJ_{E541-F680}$	Recombinant $MurJ_{E541-F680}$
C41 (DE3) pGEX Wag31	GST-Wag31 expression strain	pGEX::*wag31*	Recombinant Wag31
C41 (DE3) pGEX FhaA	GST-FhaA expression strain	pGEX::*fhaA*	Recombinant FhaA
BL21 (DE3) pGEX PknB	GST-PknB kinase domain expression strain	pGEX::*pknB kinase*	Recombinant PknB kinase domain

Table S3. Effect of threonine mutations on growth of *M. tuberculosis* in various media. Related to Figures 2 and S3.

Strain	HK 7H10 agar	HKP 7H10 agar	HK 7H9 liquid	HKP 7H9 liquid	SMM	SMP
CM-CwlM$_{pMV}$	-	+++	-	+++	-	++
CM-CwlM$_{WT}$	+++	+++	+++	+++	+++	+++
CM-CwlM$_{T43A}$	+++	+++	+++	+++	+++	+++
CM-CwlM$_{T382A}$	-	+++	-	+++	++	+++
CM-CwlM$_{T386A}$	++	+++	++	+++	+++	
CM-CwlM$_{T382A+T386A}$	No colonies	No colonies	N/A	N/A	N/A	N/A
CM-CwlM$_{T382A+T386A+T43A}$	-	+++	-	+++	++	+++
CM-CwlM$_{T42A}$	+++	+++	+++	+++	+++	+++
CM-CwlM$_{T42A+T43A}$	+++	+++	++	+++	+++	+++
CM-CwlM$_{T94A}$	+++	+++	+++	+++	+++	+++
CM-CwlM$_{T384A}$	+++	+++	+++	+++	+++	+++
CM-CwlM$_{T382A+T384A}$	-	+++	-	+++	++	+++
CM-CwlM$_{T384A+T386A}$	+++	+++	+++	+++	+++	+++
CM-CwlM $_{T382A+T384A+T386A}$	-	Small colonies	N/A	N/A	N/A	N/A
CM-CwlM$_{T43D}$	+++	+++	+++	+++	+++	+++
CM-CwlM$_{T382D}$	++	+++	++	+++	++	+++
CM-CwlM$_{T386D}$	+++	+++	+++	+++	+++	+++
CM-CwlM$_{T382D+T386D}$	-	+++	-	+++	-	+++
CM-CwlM$_{T382D+T386D+T43D}$	-	+++	-	+++	++	-
CM-CwlM$_{T42D+T43D}$	+++	+++	+++	+++	+++	+++
CM-CwlM$_{T382D+T384D}$	-	+++	-	+++	N/D	N/D
CM-CwlM$_{R2}$	-	++	-	++	-	++

N/A – not applicable; N/D – not determined; “+++” - good growth compared with WT Mtb strain; “++” or “+” growth with defect; - no growth; “HK” with addition of hygromycin and kanamycin; “HKP” with addition of hygromycin, kanamycin and pristinamycin; SMM – sucrose magnesium medium.

Table S4. Identification of potential CwlM partners by immuno-precipitation and protein fragment complementation assays. Related to Figure 4.

Protein	Gene	Function	Fraction	M-PFC
DnaA	*Rv0001*	Chromosomal replication initiator protein	Cytoplasm	N
CwsA	*Rv0008c*	Membrane protein involved in division	Membrane	Y
FhaA	*Rv0020c*	Conserved protein with FhaA	Cytoplasm	Y
MurA	*Rv1315*	UDP-N-acetylglucosamine 1-carboxyvinyltransferase	Cytoplasm	Not assessed
Wag31	*Rv2145c*	DivIVa family protein	Cytoplasm	N
FtsZ	*Rv2150c*	Cell division protein	Cytoplasm	N
FtsE	*Rv3102c*	Putative cell division ATP-binding protein	Membrane	N
MviN	*Rv3910*	Potential Lipid II flippase MurJ	Membrane	Y

N – no growth on trimethoprim plates; Y- interaction.

Table S5. Primers used in the study. Related to Figures 2-4, S2-S5.

PRIMER	SEQUENCE 5'-3'	DESCRIPTION
CMRv3915F	ACTGCCATGGGCCCGAGTCCGCGCGAA	*cwlM*-CM generation
CMRv3915R	ACGTGCATGCTCATGCGTCGGACGGACTACG	*cwlM*-CM generation
FG3106	CGTTGGCGGTGGCGGCAC	*cwlM*-CM confirmation
FG2224	CCGTACACCGTACAAGGAG	*cwlM*-CM confirmation
Rv3915pEF	ACACATATGCCGAGTCCGCGCCGCGAAGA	Recombinant CwlM
RV3915PETR1	ACAGGATCCTTAAGAACCGCCGAGTCTACCCG	Recombinant CwlM
3915PMV306F2	ACTGGTACC AGCCGGTGAAACGAATCGTT	*cwlM*-CM complementation
3915PMV306R1	TACAAGCTTTAAGAACCGCCGAGTCTACC	*cwlM*-CM complementation
3915PMV306R3	TACAAGCTTTCAAACGGCTGTATCTGTTA	*cwlM*-CM complementation
3915T43AF	CAGGAAGAAGACCTGACGGCGGGCCGTAACGTCGCCCTT	CwlM SDM
3915T43AR	AAGGGCGACGTTACGGCCCGCCGTCAGGTCTTCTTCCTG	CwlM SDM
3915T43DF	CAGGAAGAAGACCTGACGGATGGCCGTAACGTCGCCCTT	CwlM SDM
3915T43DR	AAGGGCGACGTTACGGCCATCCGTCAGGTCTTCTTCCTG	CwlM SDM
3915T382AF	GCAAGAACGATCGGCCCGCTGGCACATTCACTTTCGC	CwlM SDM
3915T382AR	GCGAAAGTGAATGTGCCAGCGGGCCGATCGTTCTTGC	CwlM SDM
3915T382DF	GGCAAGAACGATCGGCCCGACGGCACATTCACTTTCGCC	CwlM SDM
3915T382DR	GGCGAAAGTGAATGTGCCGTCGGGCCGATCGTTCTTGCC	CwlM SDM
3915T386AF	CGGCCCACCGGCACATTCGCTTTCGCCGAGTTGCTGGCC	CwlM SDM
3915T386AR	GGCCAGCAACTCGGCGAAAGCGAATGTGCCGGTGGGCCG	CwlM SDM
3915T386DF	CGGCCCACCGGCACATTCGATTTCGCCGAGTTGCTGGCC	CwlM SDM
3915T386DR	GGCCAGCAACTCGGCGAAATCGAATGTGCCGGTGGGCCG	CwlM SDM
3915T382-6AF	GCAAGAACGATCGGCCCGCTGGCACATTCGCTTTCG	CwlM SDM
3915T382-6AR	GCGAAAGCGAATGTGCCAGCGGGCCGATCGTTCTTGC	CwlM SDM
3915T382-6DF	CGGCCCGACGGCACATTCGATTTCGCCGAGTTGCTGGCC	CwlM SDM
3915T382-6DR	GGCCAGCAACTCGGCGAAATCGAATGTGCCGTCGGGCCG	CwlM SDM
3915T384AF2	ACGATCGGCCCACCGGCGCATTCACTTTCGCCGAGTT	CwlM SDM
3915T384AR2	AACTCGGCGAAAGTGAATGCGCCGGTGGGCCGATCGT	CwlM SDM
2AtailF	AACGATCGGCCCGCTGGCGCATTCACTTTCGCCGAGTTG	CwlM SDM
2AtailR	CAACTCGGCGAAAGTGAATGCGCCAGCGGGCCGATCGTT	CwlM SDM
2AendF	ACGATCGGCCCACCGGCGCATTCGCTTTCGCCGAGTT	CwlM SDM
2AendR	AACTCGGCGAAAGCGAATGCGCCGGTGGGCCGATCGT	CwlM SDM
3AtailF	AACGATCGGCCCGCTGGCGCATTCGCTTTCGCCGAGTTG	CwlM SDM
3AtailR	CAACTCGGCGAAAGCGAATGCGCCAGCGGGCCGATCGTT	CwlM SDM
T42AF	CATCAGGAAGAAGACCTGGCGACGGGCCGTAACGTCGCC	CwlM SDM
T42AR	GGCGACGTTACGGCCCGTCGCCAGGTCTTCTTCCTGATG	CwlM SDM
T42-43AF	CATCAGGAAGAAGACCTGGCGGCGGGCCGTAACGTCGCC	CwlM SDM
T42-43AR	GGCGACGTTACGGCCCGCCGCCAGGTCTTCTTCCTGATG	CwlM SDM
T94AF	TACCGGCTCGGGGCCCGCGCGCTGTACCACCAATTCGGC	CwlM SDM
T94AR	GCCGAATTGGTGGTACAGCGCGCGGGCCCCGAGCCGGTA	CwlM SDM
2DtailF	AACGATCGGCCCGACGGCGACTTCACTTTCGCCGAGTTG	CwlM SDM
2DtailR	CAACTCGGCGAAAGTGAAGTCGCCGTCGGGCCGATCGTT	CwlM SDM
T42DF	CATCAGGAAGAAGACCTGGACACGGGCCGTAACGTCGCC	CwlM SDM
T42DR	GGCGACGTTACGGCCCGTGTCCAGGTCTTCTTCCTGATG	CwlM SDM
UAB1003915F	ATAGGATCCATGCCGAGTCCGCGCCGCGAA	M-PFC
UAB1003915R	CACATCGATAGAACCGCCGAGTCTACCCGC	M-PFC

UAB200Wag31F	ACACAATTGATGCCGCTTACACCTGCCGAC	M-PFC
UAB200Wag31R	ACATCGATGTTTTTGCCCCGGTTGAATTGA	M-PFC
UAB200FhaAF	GACACAATTGATGGGTAGCCAGAAAAGGCT	M-PFC
UAB200FhaAR	AACGTCGACGTGCATGCGGACGATGATCTC	M-PFC
UAB200FtsEF	ACACAATTGATGATCACCCTGGACCATGTC	M-PFC
UAB200FtsER	GAC ATCGAT GCGATCCATCCCGTAGACGCC	M-PFC
UAB200FtsZF	ACACAATTGATGACCCCCCCGCACAACTAC	M-PFC
UAB200FtsZR	GAC ATCGATGCGGCGCATGAAGGGCGGCAC	M-PFC
UAB200DnaAF	CACAATTGTTGACCGATGACCCCGGTTCA	M-PFC
UAB200DnaAR	GAC ATCGATGCGCTTGGAGCGCTGACGGAT	M-PFC
UAB400FhaAF	ACAGAATTCGATGGGTAGCCAGAAAAGGCT	M-PFC
UAB400-FhaAR	CACAAGCTTTCAGTGCATGCGGACGATGAT	M-PFC
UAB200 MviNF1	ACACAATTGGCCGTGCGAGCCCGAATCAGGA	M-PFC
UAB200MviNR1	AACATCGATGTTGCGACGGCGGCGGGTGTA	M-PFC
UAB400MviNR1	GACATCGATCTAGTTGCGACGGCGGCGGGT	M-PFC
UAB100MviNF2	CACGTTAACATG GCCGTGCGAGCCCGAATCA	M-PFC
pETMviNF	CACCATATGGCCGTGCGAGCCCGAATCAGGA	Recombinant $MurJ_{icd}$
pETMviNR	GAC GCT AGCCTAGTTGCGACGGCGGCGGGTA	Recombinant $MurJ_{icd}$
FhaALEICF	TACTTCCAATCCATGGGTAGCCAGAAAAGGCTGGT	Recombinant FhaA
FhaALEICR	TATCCACCTTTACTGTCAGTGCATGCGGACGATGATCT	Recombinant FhaA
Wag31LeicF	TACTTCCAATCCATGCCGCTTACACCTGCCGACGT	Recombinant Wag31
Wag31LeicR	TATCCACCTTTACTGTCACTAGTTTTTGCCCCGGTTGA	Recombinant Wag31
RTPknBF1	TCAGAACGGAATCATCCACCGTGA	qRT-PCR
RTPknBR1	GCGATGCCGAAATCCATCACCTTT	qRT-PCR
PMV306F	TGGTATCTTTATAGTCCTGTC	pMV306-specific primer
pMV306R2	TAGTTAACTACGTCGACATCGA	pMV306-specific primer
MviNpETR3	CATGGATCCCTAGAACGCAATCGGCCCACG	Recombinant $MurJ_{E541\text{-}F680}$
MviNpETF2	TATCATATGGAGGCGCGGGCGGCGCTGGAT	Recombinant $MurJ_{E541\text{-}F680}$ l
