# Supplementary material for: Two Faces of CwlM, an Essential PknB Substrate, in Mycobacterium tuberculosis
Source: Cell Rep. 2018 Oct 2;25(1):57–67.e5. doi: 10.1016/j.celrep.2018.09.004 (PMC6180346; doi:10.1016/j.celrep.2018.09.004)
Supplement: Document S2. Article plus Supplemental Information [file mmc3.pdf]

Cell Reports

Article

Two Faces of CwlM, an Essential PknB Substrate, in *Mycobacterium tuberculosis*

Graphical Abstract

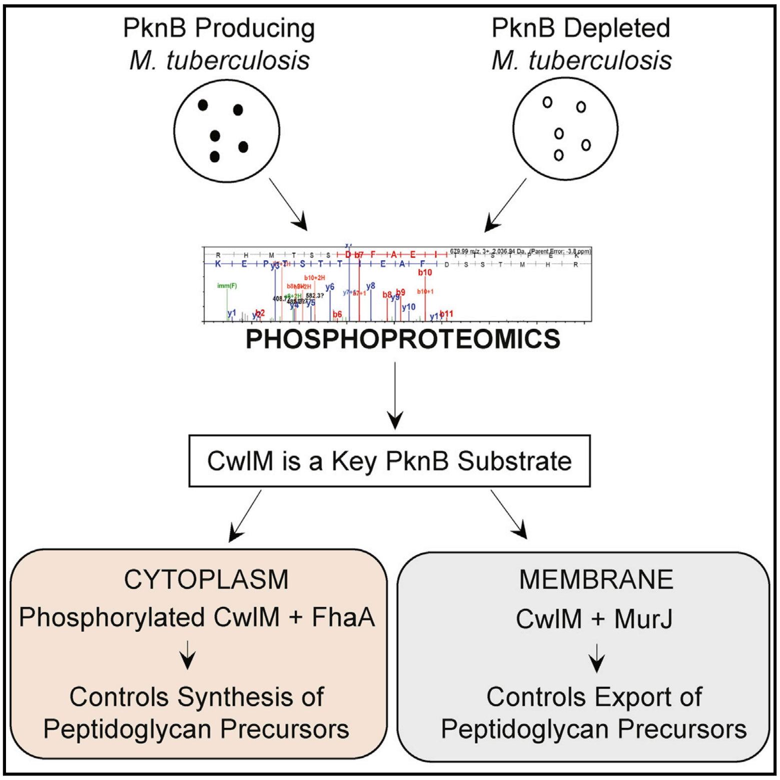

Highlights

- PknB is not critical for *M. tuberculosis* growth in osmoprotective medium
- CwlM is the major substrate of PknB
- Phosphorylation controls localization of CwlM in the cytoplasmic and membrane fractions
- Phospho-CwlM binds FhaA, and non-phospho-CwlM interacts with the essential MurJ linker

Authors

Obolbek Turapov, Francesca Forti, Baleegh Kadhim, ..., Paul Ajuh, Waldemar Vollmer, Galina V. Mukamolova

Correspondence

gvm4@le.ac.uk

In Brief

PknB controls growth and peptidoglycan biosynthesis in *Mycobacterium tuberculosis*. Turapov et al. show that CwlM, a major PknB substrate, is produced in two forms: a non-phosphorylated membrane-associated CwlM and a PknB-phosphorylated cytoplasmic CwlM. The phosphorylated CwlM binds FhaA, a fork head-associated domain protein, while non-phosphorylated CwlM interacts with MurJ (MviN), a proposed lipid II flippase.

Turapov et al., 2018, Cell Reports *25*, 57–67
October 2, 2018 © 2018 The Authors.
https://doi.org/10.1016/j.celrep.2018.09.004

OPEN ACCESS
CellPress

Two Faces of CwlM, an Essential PknB Substrate, in *Mycobacterium tuberculosis*

Obolbek Turapov,[1] Francesca Forti,[2] Baleegh Kadhim,[1,3] Daniela Ghisotti,[2] Jad Sassine,[4] Anna Straatman-Iwanowska,[5] Andrew R. Bottrill,[6] Patrick J. Moynihan,[7] Russell Wallis,[1,8] Philippe Barthe,[9] Martin Cohen-Gonsaud,[9] Paul Ajuh,[10] Waldemar Vollmer,[4] and Galina V. Mukamolova[1,11,*]

[1]Leicester Tuberculosis Research Group, Department of Infection, Immunity and Inflammation, University of Leicester, Leicester LE1 9HN, UK
[2]Department of Biosciences, University of Milan, Milan 20133, Italy
[3]Biology Department, College of Science, University of Al-Qadisiyah, Al-Diwaniyah 58002, Iraq
[4]Centre for Bacterial Cell Biology, Institute for Cell and Molecular Biosciences, Newcastle University, Newcastle upon Tyne NE2 4AX, UK
[5]Electron Microscopy Facility, Core Biotechnology Services, University of Leicester, Leicester LE1 7RH, UK
[6]Protein Nucleic Acid Laboratory, University of Leicester, Leicester LE1 7RH, UK
[7]School of Biosciences, University of Birmingham, Edgbaston, Birmingham B15 2TT, UK
[8]The Leicester Institute of Structural and Chemical Biology, Henry Wellcome Building, University of Leicester, Lancaster Road, Leicester LE1 7HB, UK
[9]Centre de Biochimie Structurale, CNRS, INSERM, University of Montpellier, Montpellier 34090, France
[10]Gemini Biosciences, Liverpool Science Park, Liverpool L3 5TF, UK
[11]Lead Contact
*Correspondence: gvm4@le.ac.uk
https://doi.org/10.1016/j.celrep.2018.09.004

SUMMARY

Tuberculosis claims >1 million lives annually, and its causative agent *Mycobacterium tuberculosis* is a highly successful pathogen. Protein kinase B (PknB) is reported to be critical for mycobacterial growth. Here, we demonstrate that PknB-depleted *M. tuberculosis* can replicate normally and can synthesize peptidoglycan in an osmoprotective medium. Comparative phosphoproteomics of PknB-producing and PknB-depleted mycobacteria identify CwlM, an essential regulator of peptidoglycan synthesis, as a major PknB substrate. Our complementation studies of a *cwlM* mutant of *M. tuberculosis* support CwlM phosphorylation as a likely molecular basis for PknB being essential for mycobacterial growth. We demonstrate that growing mycobacteria produce two forms of CwlM: a non-phosphorylated membrane-associated form and a PknB-phosphorylated cytoplasmic form. Furthermore, we show that the partner proteins for the phosphorylated and non-phosphorylated forms of CwlM are FhaA, a fork head-associated domain protein, and MurJ, a proposed lipid II flippase, respectively. From our results, we propose a model in which CwlM potentially regulates both the biosynthesis of peptidoglycan precursors and their transport across the cytoplasmic membrane.

INTRODUCTION

Tuberculosis remains a major global threat that claimed 1.3 million lives in 2016 (World Health Organization, 2017). Moreover, one-third of the entire world population is estimated to be latently infected with *Mycobacterium tuberculosis*. Multiple factors contribute to the difficulty of eradicating tuberculosis; however, the remarkable ability of *M. tuberculosis* to persist *in vivo* and to survive stressful conditions is believed to be a major contributor to the success of this pathogen (Wayne and Sohaskey, 2001). The ability of mycobacteria to adapt to varying environmental constraints is governed by numerous transcriptional, translational, and post-translational regulatory mechanisms. In particular, protein phosphorylation controls enzyme activity, protein-protein interactions, and protein localization. *M. tuberculosis* possesses 11 serine/threonine protein kinases (Prisic and Husson, 2014), two of which—protein kinase A (PknA) (Nagarajan et al., 2015) and protein kinase B (PknB) (Fernandez et al., 2006)—are essential for growth. PknB is one of the most studied mycobacterial proteins and is a verified drug target (Squeglia et al., 2017). PknB has several domains, all of which are essential for its function (Chawla et al., 2014; Prigozhin et al., 2016). The extracellular PASTA (penicillin-binding protein and serine/threonine kinase associated) domain is believed to recognize peptidoglycan fragments, and it has been implicated in PknB localization (Yeats et al., 2002; Mir et al., 2011), while the juxtamembrane domain recruits FhaA (Roumestand et al., 2011) and possibly other proteins that control peptidoglycan biosynthesis. PknB has been shown to phosphorylate multiple substrates, including proteins involved in peptidoglycan biosynthesis and remodeling: PonA1 (Kieser et al., 2015), GlmU (Parikh et al., 2009), MviN (Gee et al., 2012), and CwlM (Boutte et al., 2016). In addition, PknB interacts with Mur ligases (Munshi et al., 2013) and proteins associated with lipid metabolism (Wu et al., 2017). However, the reason for PknB essentiality is currently unknown.

Here, we present multiple facts and results that demonstrate that PknB-depleted *M. tuberculosis* can survive and replicate in osmoprotective medium, suggesting that under these

 © 2018 The Authors.
This is an open access article under the CC BY license (http://creativecommons.org/licenses/by/4.0/).

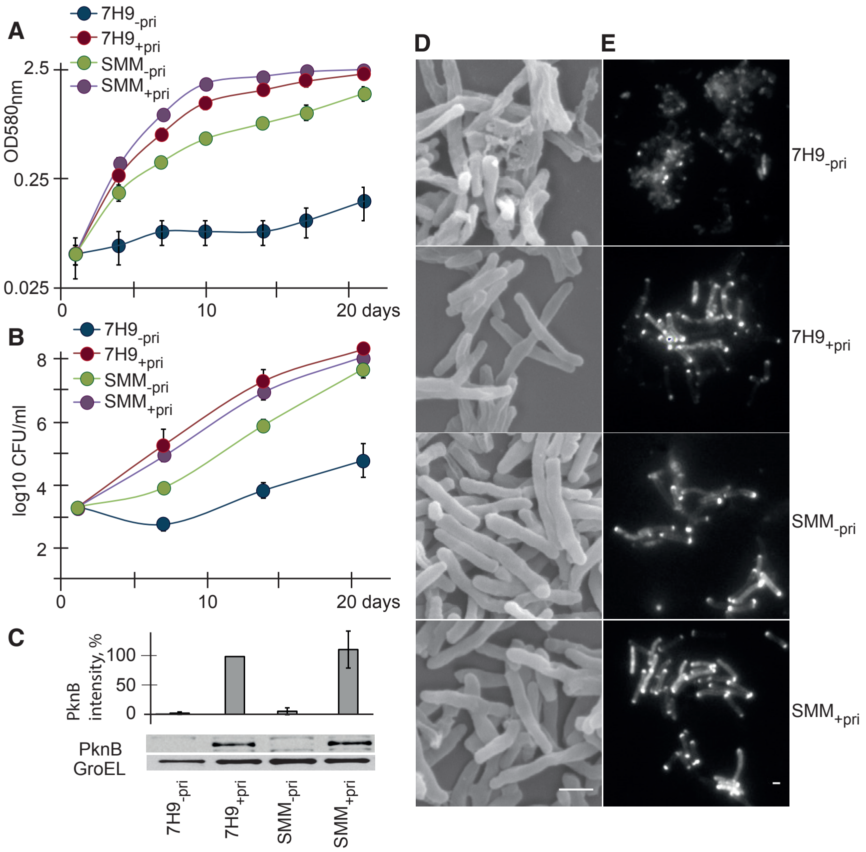

Figure 1. Osmoprotective Medium Supports Growth of a Conditional *pknB* Mutant

(A–E) *M. tuberculosis* mutant was grown in standard 7H9 medium with (7H9$_{+pri}$) or without (7H9$_{-pri}$) pristinamycin or in sucrose-magnesium medium with (SMM$_{+pri}$) or without (SMM$_{-pri}$) pristinamycin at 37°C with shaking. Growth was monitored by (A) measurement of optical density at 580 nm and by (B) assessment of colony-forming unit (CFU) counts on 7H10 agar. Data are represented as means ± SEMs (n = 6).

(C) PknB was detected using anti-PknB antibody; relative intensity of PknB bands presented as means ± SEMs (n = 3).

(D) Scanning electron micrographs of *M. tuberculosis* bacteria.

(E) Detection of nascent peptidoglycan by Van-BODIPY labeling. Scale bars, 1 μm.

conditions, PknB is not critical for bacterial growth and division. Our findings confirm that CwlM is a major substrate of PknB and demonstrate that phosphorylation determines both the cellular localization and molecular interactions of CwlM in the control of peptidoglycan biosynthesis.

RESULTS

Osmoprotective Medium Supports Growth of PknB-Depleted *M. tuberculosis*

According to previously published data, PknB depletion leads to the cessation of mycobacterial growth and to mycobacterial lysis (Kang et al., 2005; Forti et al., 2009), thus precluding any systematic analysis using omics technologies. To overcome this challenge, we developed a special osmoprotective medium and used it to investigate the growth and survival of the previously described *pknB* conditional mutant of *M. tuberculosis*, *pknB*-CM (Forti et al., 2009). In our experiments, the conditional mutant grew in standard 7H9 medium supplemented with pristinamycin, the inducer of *pknB* expression, while the omission of pristinamycin resulted in growth inhibition and in the accumulation of lysed bacteria, consistent with the previous analysis (Forti et al., 2009) (Figures 1A, 1B, and 1D). Osmoprotective sucrose-magnesium medium (SMM) not only prevented the lysis of the mutant but also supported its growth, even without pristinamycin (Figures 1A and 1B). Western blot analysis using anti-PknB antibody confirmed that PknB was depleted to <5% of the original level in media lacking pristinamycin (Figure 1C), and qRT-PCR analysis showed that *pknB* expression was indeed downregulated 8.6 ± 0.6-fold in pristinamycin-depleted cultures (SMM$_{-pri}$) compared with pristinamycin-supplemented bacteria (SMM$_{+pri}$). SMM$_{-pri}$ *pknB*-CM cells retained pristinamycin-dependent growth in standard media, so they were not escape mutants with uncontrolled *pknB* expression. Furthermore, SMM itself did not significantly influence the growth of *pknB*-CM in the presence of pristinamycin (Figure 1). Thus, our results demonstrated that the *pknB*-depleted *M. tuberculosis* bacilli were able to survive and grow in SMM. Although the bacteria had a minor growth defect under these conditions, they still reached stationary phase. The *pknB*-CM did not grow on solidified SMM without pristinamycin. *PknB*-CM cells grown in liquid SMM were slightly swollen but showed no significant cellular damage in scanning electron micrographs (Figure 1D), in contrast to *pknB*-CM bacteria grown in standard medium without pristinamycin. Notably, SMM$_{-pri}$ cells were distinct from the L-forms described for various bacterial species (Errington et al., 2016) and had a properly formed cell envelope (Figure 1D). To investigate peptidoglycan biosynthesis in the *pknB*-CM, we performed BODIPY FL vancomycin-labeling experiments. Vancomycin binds to the D-alanine-D-alanine component of nascent peptidoglycan and is used to label the mycobacterial cell wall (Joyce et al., 2012; Gee et al., 2012). The *pknB*-CM cells grown in SMM$_{-pri}$ were able to bind BODIPY FL vancomycin, indicating the production of nascent peptidoglycan (Figure 1E).

In summary, our osmoprotective medium supports the growth of PknB-depleted mycobacteria, thus providing a useful tool for investigating the role of PknB in mycobacterial biology and enabling us to conduct phosphoproteomic analyses of PknB-producing and PknB-depleted *M. tuberculosis*.

Table 1. Peptides with Increased Phosphorylation in PknB-Producing *M. tuberculosis*

Protein	Gene	Function	Essential Y/N	Identified Phosphopeptides[a]	Fold Change
PknB	*Rv0014c*	serine/threonine protein kinase	Y	TSLLSSAAGNLSGPR**T**DPLPR	33.50
				AIADSGNSVTQ**T**AAVIGTAQYLSPEQAR	10.40
				AIAD**S**GNSVTQTAAVIGTAQYLSPEQAR	6.11
UvrA	*Rv1638*	exonuclease	N	FLAEVVGGGASAA**T**SR	5.21
EspI	*Rv3876*	secretion protein EspI	N	RVHPDLAAQHAAAQPD**S**ITAA**T**TGGR	4.43
				VHPDLAAQHAAAQPDSI**T**AA**T**TGGR	2.2
Rv2406c	*Rv2406c*	conserved protein	N	MGELEAEQQQLQ**S**YITQG	3.9
CwlM	*Rv3915*	*N*-acetyl-muramyl-L-alanine amidase homolog	Y	NDRP**T**GTFTFAELLAHELSVER	3.83
				NDRPTGTF**T**FAELLAHELSVER	3.82
RodA	*Rv0017c*	cell division protein	N	SPITAAG**T**EVIERV	3.03
Lsr2	*Rv3597*	H-NS-like protein	AG[b]	IPADVIDAYHAA**T**	2.53
TrxB1	*Rv1471*	thioredoxin	N	AYEVEAGEAT**T**QNGR	2.45
CysA1	*Rv2397c*	ABC transporter	AG	GG**T**EAGNLATSMMK	2.38
EthR	*Rv3855*	transcription repressor	N	TTSAA**S**QASLPR	2.29
RpsC	*Rv0707*	ribosomal protein	Y	AAGGEEAAPDAAAPVEAQSTE**S**	2.28
FadE10	*Rv0873*	acyl-CoA de hydrogenase	N	AQQTQV**T**EEQAR	2.27
Rv2908c	Rv2908c	hypothetical protein	AG	**S**AVVVDAVEHLVR	2.03

CoA, coenzyme A. See also Table S1 and Figure S1.
[a]Phosphorylated residues are shown in bold font.
[b]AG, advantageous for growth.

PknB Depletion Leads to Global Changes in Protein Phosphorylation

A comparative analysis of phosphoproteins from PknB-depleted (SMM_{-pri}) and PknB-producing (SMM_{+pri}) *M. tuberculosis* cultures revealed global changes in phosphorylation patterns (Table S1). The depletion of PknB resulted in the increased phosphorylation of various proteins, including serine/threonine protein kinase PknA. Other abundant phosphoproteins in the SMM_{-pri} cultures were ribosomal proteins, heat shock proteins, transporters, and factors involved in cell division. Several previously annotated PknB substrates such as MurJ (also known as MviN) (Gee et al., 2012), FhaA (Roumestand et al., 2011), and GarA (Villarino et al., 2005) that control peptidoglycan biosynthesis and central metabolism showed increased phosphorylation in the PknB-depleted samples (Table S1). In addition, phosphorylated MtrA and PrrA, two component response regulators essential for *M. tuberculosis* growth (Zahrt and Deretic, 2000; Haydel et al., 2012), were more abundant in the PknB-depleted cultures.

To identify potential PknB-specific substrates, we analyzed phosphopeptides that were enriched in SMM_{+pri} cultures relative to SMM_{-pri} cultures. In total, 13 proteins were found to be >2-fold more phosphorylated in SMM_{+pri} samples; 6 of them had been previously annotated as proteins essential or advantageous for *M. tuberculosis* growth (DeJesus et al., 2017) (Tables 1 and S1). As expected, PknB was the most phosphorylated protein in PknB-producing *M. tuberculosis.* Other substrates with increased phosphorylation included CwlM, a peptidoglycan amidase homolog (Boutte et al., 2016); the annotated enzymes, UvrA, an exonuclease (Rossi et al., 2011), and FadE10, an acyl-dehydrogenase; transcriptional regulators Lsr2 (Bartek et al., 2014) and EthR (Leiba et al., 2014); an RNA-binding protein, RpsC, which is involved in translation initiation; secretion and membrane proteins EspI (Zhang et al., 2014) and Rv2397c ABC transporter; and conserved proteins of unknown function, Rv2406c and Rv2908. Most proteins had one phosphosite; however, PknB itself, CwlM, and EspI were phosphorylated on several amino acids.

Among the PknB substrates showing increased phosphorylation, PknB, CwlM, and RpsC represented potential PknB substrates essential for growth. In particular, CwlM, encoded by *rv3915*, was the most highly phosphorylated essential protein (after PknB itself) in the PknB-producing *M. tuberculosis* compared with the PknB-depleted mycobacteria. We therefore focused our investigation on this target. Four phosphosites were detected in CwlM (threonine 42 [T42], T43, T382, and T386); however, only two of these (T382 and T386) were more phosphorylated in the PknB-producing mycobacteria (Table 1; Figure S1).

PknB was able to phosphorylate *M. tuberculosis* CwlM *in vitro* (Figure S1). Mass spectrometry analysis of *in vitro* phosphorylated CwlM confirmed phosphorylation of T43, T382, and T386, and identified two additional phosphorylated residues, T94 and T384. Similarly, Boutte et al. (2016) have recently reported that PknB phosphorylates *M. tuberculosis* CwlM *in vitro*. The biological importance of CwlM phosphorylation at these sites was further investigated in complementation studies.

A Phosphoablative CwlM Mutant of *M. tuberculosis* Mimics the Phenotype of PknB-Depleted Mycobacteria

We reasoned that if CwlM is the main substrate of PknB, a phosphoablative mutant of CwlM should reproduce the major features of the PknB-depleted mycobacteria. We first generated a *cwlM* conditional mutant of *M. tuberculosis* (*cwlM*-CM) using the

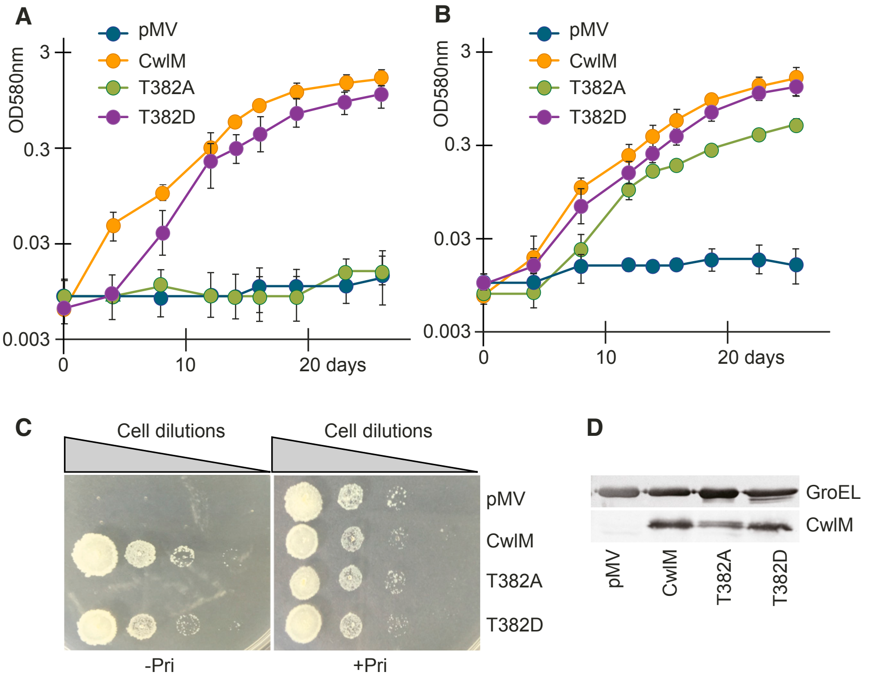

Figure 2. T382A Mutant Mimics Phenotype of PknB-Depleted *M. tuberculosis*

(A–D) The *cwlM* conditional mutant of *M. tuberculosis* was transformed with pMV306 plasmids containing *cwlM* variants. The resultant strains were grown in 7H9 medium (A) or in SMM (B) without pristinamycin. All of the strains grew similarly when 7H9 or SMM were supplemented with pristinamycin (data not shown for clarity). pMV, *cwlM*-CM$_{pmv306}$ (the empty plasmid control); CwlM, *cwlM*-CM$_{WT}$; T382A and T382D phosphoablative and phosphomimetic mutants, respectively. Data are represented as means ± SEMs (n = 6).

(C) Growth of strains on 7H10 agar.

(D) Western blot of CwlM variants detected with anti-CwlM antibody.

See also Figures S2 and S3 and Table S3.

pristinamycin-inducible system. As expected, the mutant did not grow without pristinamycin in liquid (Figures S2A and S2B) or on solid (Figure S2C) media. Western blot analysis using a CwlM-specific antibody confirmed the near-complete depletion of CwlM in the *cwlM*-CM mutant upon the withdrawal of pristinamycin (Figure S2D). CwlM depletion resulted in severe cell aggregation, the accumulation of lysed mycobacteria (Figure S2D), and the cessation of BODIPY FL vancomycin incorporation (Figure S2F).

This dramatic phenotype could be fully complemented by the reintroduction of *cwlM* with a putative upstream promoter in an integrating plasmid, pMV306. The complemented mutant (*cwlM*-CM$_{WT}$) was able to grow in liquid and solid media without pristinamycin, while a strain with an empty pMV306 plasmid (*cwlM*-CM$_{pmv306}$) displayed the CwlM depletion phenotype (Figure 2). A panel of site-directed mutants was generated (Table S2) to study the importance of phosphorylation at the different threonine sites. The growth patterns of the resultant *M. tuberculosis* strains are summarized in Table S3. Single replacements of T42, T43, T94, T384, or T386 (Figure S3A) with an alanine did not have a significant effect on *M. tuberculosis* growth. However, mycobacteria expressing the T382A variant could not grow without pristinamycin either in liquid medium (Figure 2A) or on agar (Figure 2C), highlighting the T382A mutation as being critical for growth. The replacement of T382 with an aspartate residue (T382D) to mimic phosphorylation resulted in a milder growth defect (Figures 2A and 2C), while replacement of any other phosphosites with an aspartate had no marked effect on *M. tuberculosis* growth (Table S3; Figure S3).

A double phosphoablative mutation (T382A and T386A) was very toxic to *M. tuberculosis*, and no transformants could be recovered with this construct. The corresponding double phosphomimetic mutation (T382D+T386D) was not toxic for mycobacteria, but it did not complement the mutant phenotype (Figure S3B). These findings suggest that phosphorylation of both threonines is important for *M. tuberculosis* growth.

We next investigated whether the phosphoablative T382A version of *cwlM*-CM could grow in osmoprotective medium. *CwlM*-CM and *cwl*M-CM$_{pmv306}$ strains did not grow in SMM (Figure 2B), while the *cwlM*-CM$_{WT}$ and the T382D phosphomimetic grew similarly in standard and SMM (Figures 2A and 2B). Furthermore, the T382A variant was able to grow in SMM$_{-pri}$ (Figure 2B) and to incorporate BODIPY FL vancomycin (data not shown), thus mimicking the phenotype of the PknB-depleted *M. tuberculosis*. As demonstrated in Figure 2D, all of the variant proteins were produced at similar levels. Attempts to complement the *pknB*-CM mutant with any of the phosphomimetic forms were unsuccessful.

CwlM Is Present in Two Distinct Forms during Mycobacterial Growth

The phenotypes of PknB-depleted and CwlM-depleted mycobacteria, as well as those of the phosphoablative and phosphomimetic CwlM *M. tuberculosis* mutants in the present study, suggest that both phosphorylated and non-phosphorylated forms of CwlM play important roles in mycobacterial growth. We hypothesized that the phosphorylated and non-phosphorylated forms may have different cellular localizations. To test our hypothesis, we performed cell fractionation for western blot analysis and investigated the presence of CwlM in *cwlM*-CM and *pknB*-CM samples. As shown in Figure 3, CwlM was detected in both the cytoplasmic and membrane fractions of *cwlM*-CM$_{WT}$ (Figure 3A, wild-type [WT]) and of pristinamycin-induced *pknB*-CM (Figure 3B, 7H9$_{+pri}$ and SMM$_{+pri}$). Similar results were obtained in WT *M. tuberculosis* and *M. smegmatis* (Figure S4). Both forms were missing in the control *cwlM*-CM$_{pmv306}$ grown without pristinamycin (Figure 3A). The T382A form was present in the membrane fraction but not in the cytoplasmic fraction, while the T382D phosphomimetic was present in both fractions, with a slight reduction in the membrane fraction. Furthermore, PknB

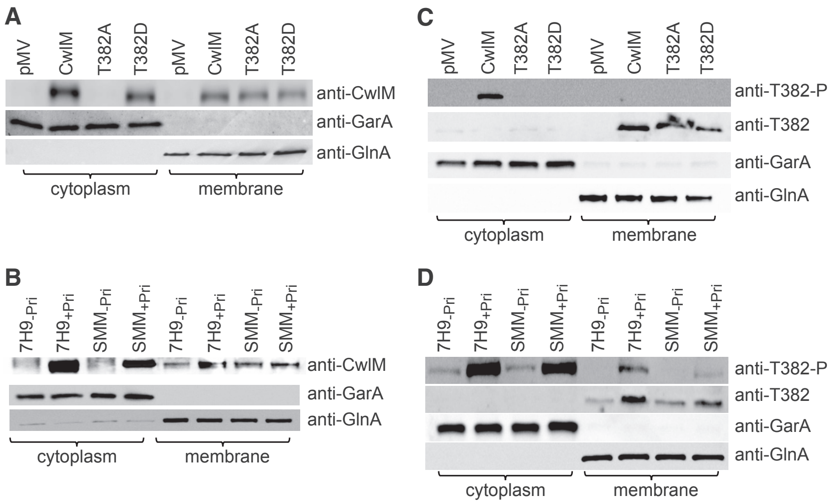

Figure 3. PknB-Mediated Phosphorylation of T382 Determines Distribution of CwlM in Cytoplasmic and Membrane Fractions of *M. tuberculosis*

(A–D) Lysates obtained from *cwlM*-CM grown in SMM without pristinamycin to prevent induction of genomic *cwlM* (A and C) or from *pknB*-CM grown in SMM or standard 7H9 medium with or without pristinamycin (B and D) were fractionated and probed with anti-CwlM antibodies (A and B) or with anti-T382-P and anti-T382 antibodies (C and D). Anti-GarA and Anti-GlnA antibodies were used to confirm the purity of mycobacterial fractions.

See also Figure S4.

depletion resulted in the loss of cytoplasmic but not of membrane CwlM (Figure 3B). Both forms of CwlM were detectable only during the exponential growth phase (Figure S4).

Our complementation data suggested that the phosphorylation of T382 by PknB is critical for *M. tuberculosis* growth in standard media. To detect the phosphorylation state of CwlM in *M. tuberculosis* fractions, we generated phosphosite-specific antibodies. Two different antibodies were used. The first antibody was raised against a peptide containing the phosphorylated form of T382, designated as anti-T382-P antibody, and the second was raised against an equivalent non-phosphorylated peptide, designated as anti-T382 antibody. Western blot analysis using anti-T382-P and *M. tuberculosis* lysates indicated that phosphorylated CwlM was present only in the cytoplasm of *cwlM*-CM$_{WT}$ (Figure 3C). The T382A and T382D mutants were not phosphorylated, while *cwlM*-CM$_{pmv306}$ did not produce CwlM. Consistent with these findings, non-phosphorylated CwlM was not detected in the cytoplasm, but instead WT T382, T382A, and T382D forms were present in the membrane fractions (Figure 3C). Furthermore, the T382 phosphorylated CwlM was mainly detected in the cytoplasm of pristinamycin-induced *pknB*-CM, but it was significantly reduced in PknB-depleted mycobacteria (Figure 3D). Non-phosphorylated CwlM was detected in the membrane fractions of *pknB*-CM under all of the conditions tested (Figure 3D). These results suggest that in growing bacteria, CwlM is present as both phosphorylated and non-phosphorylated forms. The cytoplasmic form is phosphorylated, whereas the membrane-associated form is non-phosphorylated. In addition, PknB controls the distribution of CwlM via the phosphorylation of T382.

Phosphorylated and Non-phosphorylated CwlM Have Different Protein Partners

It has recently been reported that T382-phosphorylated CwlM interacts with MurA, which is located in the cytoplasm and is the first enzyme in the biosynthesis of peptidoglycan precursors, and stimulates its activity (Boutte et al., 2016). Given that CwlM has no predicted transmembrane domains or lipid anchors, it was reasonable to hypothesize that the membrane-associated non-phosphorylated form may interact with other membrane protein(s). To test this possibility, we prepared membrane and cytoplasmic *M. tuberculosis* fractions for immunoprecipitation assays using the anti-CwlM antibody. Several potential partners of CwlM were identified in cytoplasmic and membrane fractions (Table S4). These partners included FhaA, FtsZ, DnaA, Wag31, and the previously described MurA in the cytoplasmic fraction, and MurJ (MviN), FtsE, and CwsA in the membrane fraction.

Mycobacterial protein fragment complementation assays further confirmed that CwlM interacts with FhaA, MurJ, and CwsA (Figure S5). MurJ is an integral membrane protein with proposed lipid II flippase activity based on cellular assays (Sham et al., 2014). Although the purified protein is reported to lack lipid II transport activity (Mohammadi et al., 2011), lipid II binding to MurJ has been detected by native mass spectrometry and factors that influence the interaction of MurJ with lipid II identified (Bolla et al., 2018). Mycobacterial MurJ is characterized by unique structural properties; in addition to 14 highly conserved transmembrane helices, it has an intracellular domain of 334 amino acids, designated as MurJ$_{icd}$ (Gee et al., 2012). We considered MurJ$_{icd}$ as the likely CwlM-binding domain and focused our investigation on this domain rather than on the entire protein.

FhaA contains a C-terminal fork head-associated (FHA) domain that interacts with phosphorylated proteins (Roumestand et al., 2011). MurJ$_{icd}$ and FhaA have been previously shown to interact with each other (Gee et al., 2012), and we were intrigued by the possibility that CwlM may interact with both proteins. We therefore generated recombinant FhaA and MurJ$_{icd}$ and investigated their interaction with both forms of CwlM. Recombinant Wag31 was used as a control. As Figure 4 shows, Wag 31 did not co-precipitate with either CwlM forms, while MurJ$_{icd}$ mainly co-precipitated with non-phosphorylated CwlM. Densitometric analysis of gels from three independent experiments confirmed that 85% $\pm$ 6% of MurJ$_{icd}$ was co-precipitated with non-phosphorylated CwlM compared with 12% $\pm$ 6% bound to phosphorylated CwlM. FhaA showed the opposite binding pattern, with 84% $\pm$ 4% co-precipitating with phosphorylated CwlM and only 19.7% $\pm$ 9% with non-phosphorylated CwlM. As shown in Figure 4D, the CwlM bound to FhaA was phosphorylated on T382, while the CwlM co-immunoprecipitated with MurJ$_{icd}$ was not phosphorylated.

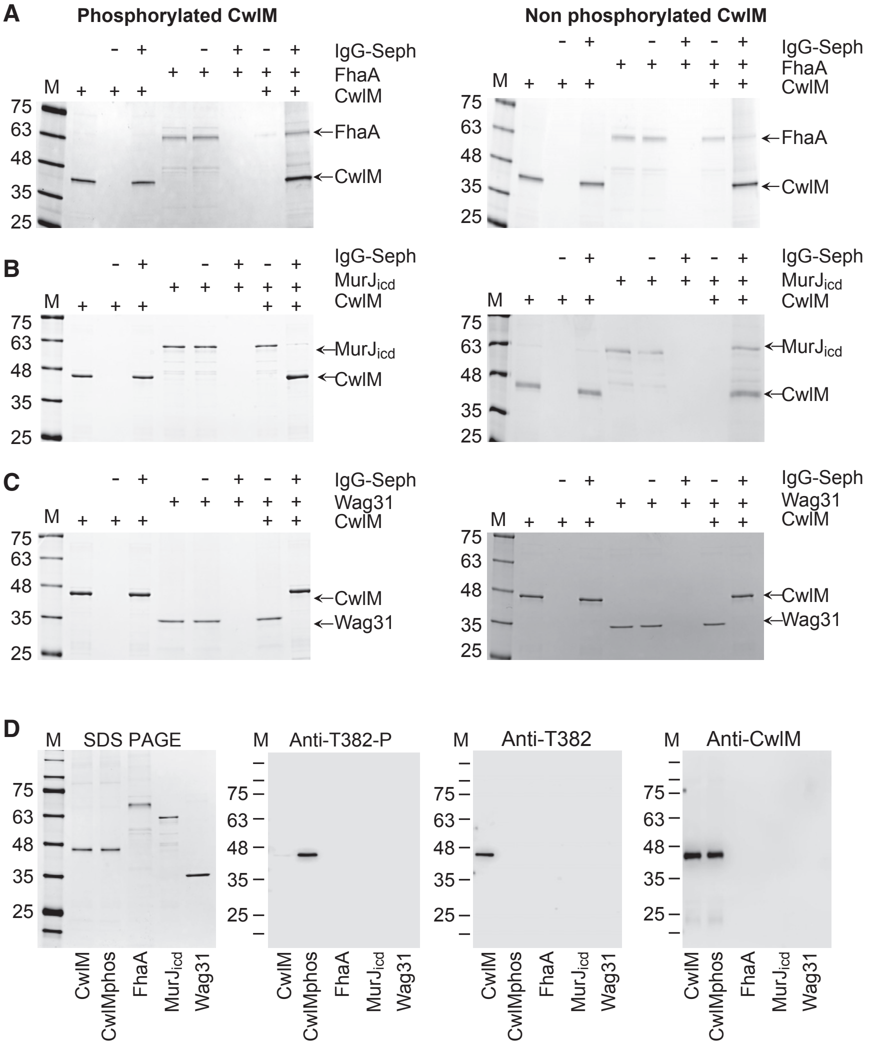

Figure 4. PknB Phosphorylation Controls the Interaction of CwlM with $MurJ_{ICD}$ and FhaA

(A–D) PknB-phosphorylated and non-phosphorylated recombinant CwlM was mixed with recombinant FhaA (A), $MurJ_{ICD}$ (B), or Wag31 (C) and incubated with gentle mixing for 30 min. Anti-CwlM immunoglobulin G (IgG) Sepharose (IgG-Seph) was then added and further incubated for 30 min. Proteins bound to Sepharose and unbound material were resolved on SDS-PAGE and stained with Coomassie brilliant blue.

(D) Confirmation of CwlM phosphorylation. M, protein markers; +, reagent added; −, flow-through fractions.

See also Figures S5 and S7 and Table S4.

CwlM Binding to FhaA Is Driven by T382 Phosphorylation and Increased by T386 Phosphorylation

Our phosphoproteomics and complementation studies highlighted the importance of T382 and T386 phosphorylation for *M. tuberculosis* growth. We therefore explored the role of these phosphosites for binding to FhaA, using the recombinant C-terminal domain of FhaA (designated as FHA) and synthetic peptides corresponding to the C-terminal tail of CwlM. These included single phospho-T382 and phospho-T386 peptides, a double phospho-T382 and phospho-T386-peptide, a non-phosphorylated peptide, a double phosphomimetic (T382D and T386D), and double phosphoablative peptides (T382A and T386A). Using two-dimensional nuclear magnetic resonance spectroscopy, we detected chemical shifts in the [^{1}H,^{15}N] heteronuclear single quantum coherence (HSQC) spectra of the ^{15}N-labeled FHA domain upon the addition of all of the phosphopeptides, indicating binding (Figure 5). Differences were observed in the pattern of chemical shift changes in FHA for the two single phosphopeptides. For example, the chemical shifts of T470 and G471 were appreciably more perturbed upon the addition of the T382 phosphopeptide than upon addition of the T386 phosphopeptide, despite the presence of two phenylalanines in positions 385 and 387. This observation indicates that the T386 phosphopeptide may bind more weakly to FHA compared to the binding of the T382 peptide. Perturbations in the [^{1}H,^{15}N] HSQC spectra of the ^{15}N labeled FHA domain, observed upon the addition of the double phosphopeptide, corresponded to those observed for the single T382 phosphopeptide (Figure S6) but with larger changes in the chemical shifts. T470 and T471 of FHA were again perturbed. These findings suggest that the primary binding site in FHA was occupied by phospho-T382 and that T386 phosphorylation played an accessory role by increasing this interaction but without replacing the phospho-T382 as the main anchor for FHA binding. The additional phosphorylation of other FHA domains has been reported to have a similar effect on protein-protein interactions (Lee et al., 2008). In control experiments, we tested non-phosphorylated and double phosphoablative peptides and did not observe any chemical shift in the FHA spectra, while the double phosphomimetic peptide displayed a weaker binding. The role of T384 phosphorylation in FHA and CwlM interaction was not investigated.

Thus, we can postulate that phosphorylation at the T382 position is critical for the interaction of CwlM with FhaA and that additional phosphorylation potentiates the binding of these two proteins. These data further support our mutant complementation results (Figure S3; Table S3), which together indicate that phosphorylated threonines have distinct roles in CwlM function.

Non-phosphorylated CwlM Interacts with an Essential Part of $MurJ_{icd}$

We hypothesized that the interaction of CwlM with MurJ could be essential for mycobacterial growth. According to Gee et al. (2012), not all MurJ domains are essential for *M. tuberculosis*

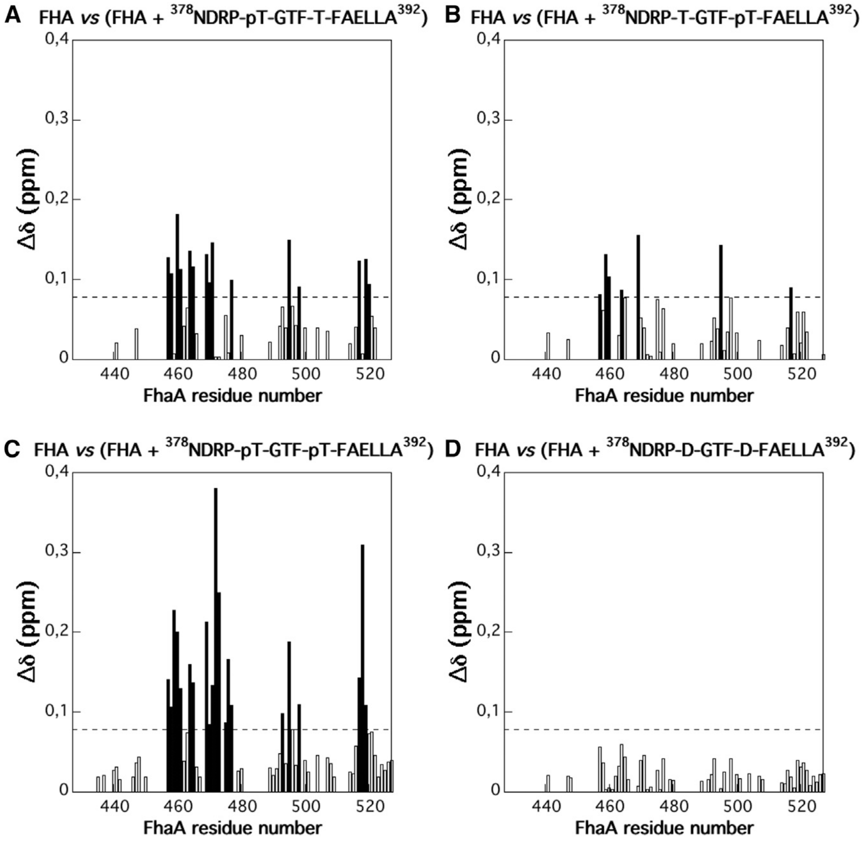

Figure 5. Amide Averaged Chemical Shift Variations (Δδ) as a Function of Protein Sequence

(A–D) Δδ values were calculated between ^{1}H-^{15}N HSQC spectra recorded at 800 MHz (20°C and pH 6.8) on 80 μM ^{15}N-uniformly labeled samples of Rv0020c-FHA before and after addition of 80 μM concentrations of unlabeled peptides pT382–T386 (A), T382–pT386 (B), pT382–pT386 (C), or D382–D386 (D), with $\Delta\delta = [(\Delta\delta_H)^2 + (\Delta\delta_N \times (\gamma_N/\gamma_H))^2]^{0.5}$. The dotted lines show the SD (0.078 ppm) from the "C" position.

See also Figure S6.

viability. These authors obtained viable deletion mutants when the protein was truncated at phenylalanine 715; however, shorter truncated MurJ forms did not support mycobacterial growth. These results indicated that the E541–F680 region of $MurJ_{icd}$, which links the 14th transmembrane helix with the pseudokinase domain, may be indispensable for mycobacterial growth, while the non-essential pseudokinase domain (D681–R963) may have a regulatory role via the recruitment of the FHA domain of FhaA (Gee et al., 2012). No function has been described for the E541–F680 region. We therefore tested whether this region can bind to CwlM. We generated a recombinant version of this linker for use in immunoprecipitation experiments. As shown in Figure S7, the linker did indeed bind CwlM. Our attempts to generate a mycobacterial mutant lacking this region were unsuccessful, confirming previously published results on the essentiality of this part of $MurJ_{icd}$ (Gee et al., 2012). Thus, we established that non-phosphorylated CwlM interacts with an essential region of MurJ. The lack of BODIPY FL vancomycin labeling in the CwlM-depleted mycobacteria (Figure S2) indicates that this interaction may be important for the production of nascent peptidoglycan.

DISCUSSION

PknB Is Not Critical for *M. tuberculosis* Growth in Osmoprotective Medium

PknB-like kinases are widely distributed in Gram-positive bacteria (Pereira et al., 2011). Most are not essential for bacterial viability and they fulfill distinct biological functions. For example, in *Bacillus subtilis*, PrkC is not required for growth and regulates spore germination (Shah et al., 2008), while in *Streptococcus pneumoniae*, StkP is important for cell division and cell wall remodeling (Beilharz et al., 2012; Zucchini et al., 2018). It is widely accepted that PknB is essential for mycobacterial viability (Fernandez et al., 2006) because of its involvement in regulating peptidoglycan biosynthesis and cell shape (Kang et al., 2005). PknB is produced during exponential growth and its altered expression dramatically affects mycobacterial growth and morphology. PknB depletion leads to the accumulation of elongated cells and to gradual bacterial lysis (Forti et al., 2009), while *pknB* overexpression affects cell viability and morphology (Kang et al., 2005). These effects of dysregulated PknB expression on bacterial viability have precluded a detailed molecular analysis of its essentiality for mycobacterial viability, given that altered phosphoproteomics profiles could be attributed to "dying cells."

In this study, we developed a special medium that prevented the death of PknB-depleted mycobacteria and supported their propagation. The PknB-depleted *M. tuberculosis* bacilli were able to synthesize peptidoglycan and showed only marginal changes in morphology. The need for an osmoprotective medium suggests that the PknB-depleted mutant has defects in peptidoglycan structure and that PknB has a regulatory role in peptidoglycan biosynthesis. We have previously shown that the overexpression of the PknB_PASTA domain partially mimics the phenotypes of *pknB*-depleted mycobacteria by inhibiting mycobacterial growth and causing increased sensitivity to meropenem (Turapov et al., 2015), the inhibitor of transpeptidases and D,D-carboxypeptidase in mycobacteria (Kumar et al., 2012). The inhibition of PknB-like kinases in other bacteria also increases bacterial susceptibility to β-lactam antibiotics (Vornhagen et al., 2015; Pensinger et al., 2018), indicating that these kinases may control peptidoglycan biosynthesis. We therefore propose that PknB depletion could result in defective peptidoglycan synthesis, which is incompatible with growth in standard conditions.

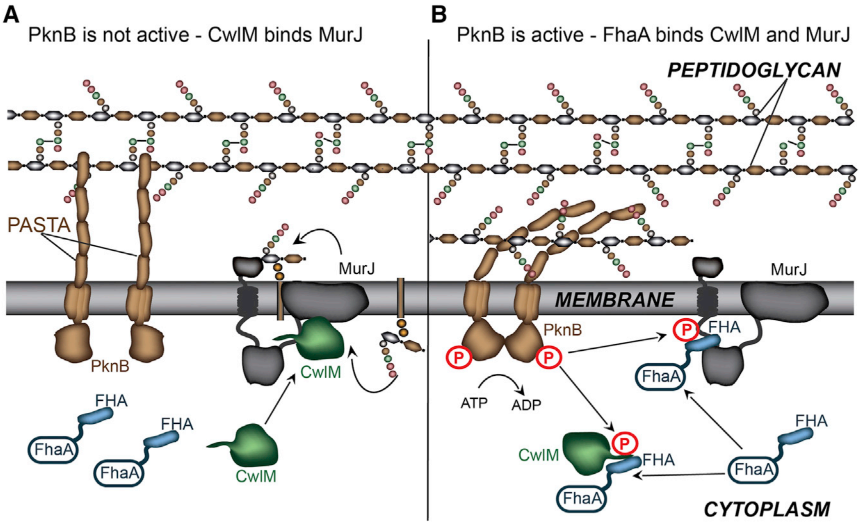

Figure 6. Proposed CwlM-Mediated Regulation of Peptidoglycan Synthesis in Mycobacteria

(A) In this model, non-phosphorylated CwlM interacts with the essential MurJ linker region and activates or facilitates the transport of peptidoglycan precursors. This activity may lead to the accumulation of excessive amounts of peptidoglycan, which is not incorporated into the cell wall.
(B) The PASTA domain of PknB senses un-crosslinked peptidoglycan, resulting in the autophosphorylation and activation of PknB. PknB then phosphorylates CwlM and MurJ, which both interact with FhaA. Phosphorylated CwlM also interacts with MurA (not included for clarity). FhaA may serve as a regulatory hub to ensure that a balance is maintained between the phosphorylated and non-phosphorylated forms of CwlM and that interactions between CwlM and its partners, MurJ and MurA, are regulated. The red P shows phosphorylation of PknB, CwlM, and MurJ.

CwlM Is a Major PknB Substrate

A substantial number of PknB substrates have been identified using *in vitro* phosphorylation assays (Prisic and Husson, 2014). Several phosphoproteomics studies have also demonstrated a high abundance of phosphoproteins in mycobacteria (e.g., Prisic et al., 2010); however, there is limited information about the specific kinases that are responsible for the phosphorylation of these proteins. One study attempted to identify PknB substrates in a strain overexpressing PknB at early stationary phase (Kang et al., 2005), while a more recent investigation analyzed phosphopeptides from *M. tuberculosis* treated with kinase inhibitors compared to untreated controls (Carette et al., 2018).

Here, we conducted a comparative phosphoproteomics analysis of PknB-producing and PknB-depleted mycobacteria in the exponential growth phase and identified potential PknB substrates. This analysis identified CwlM as being the strongest candidate for a main PknB substrate. CwlM is essential for growth and is likely to be involved in the regulation of cell wall biosynthesis. A recent study (Boutte et al., 2016) reported that PknB phosphorylates CwlM *in vitro* and that a T374 phosphoablative CwlM mutant of *M. smegmatis* had a severe growth defect in liquid and solid media. These authors' genetic and biochemical evidence suggests that phosphorylated CwlM stimulates the activity of MurA, the first enzyme in the biosynthesis of peptidoglycan precursors, and that it is therefore likely to be directly involved in the regulation of peptidoglycan precursor production. Our findings confirm that this regulation is not essential under osmoprotective conditions.

Our study also demonstrated that the phosphorylation of T382 in CwlM is critical for *M. tuberculosis* growth in standard but not in osmoprotective media. This remarkable similarity between the phenotypes of PknB-depleted and phosphoablative-CwlM mycobacteria, together with the direct demonstration of dramatically decreased levels of phosphorylated CwlM in the PknB-depleted strain, suggests that CwlM phosphorylation may explain why PknB is essential for *M. tuberculosis* viability. However, the phosphorylation of other PknB substrates may also be critical for *M. tuberculosis* viability under certain conditions.

Phosphorylated and Non-phosphorylated CwlM Proteins Have Distinct Cell Localizations and Different Protein Partners

Our results indicate that both phosphorylated and non-phosphorylated forms of CwlM have distinct roles in *M. tuberculosis* growth. CwlM-depleted mycobacteria cannot neither grow in osmoprotective SMM nor incorporate BODIPY FL vancomycin, while the phosphoablative mutant can grow in SMM and incorporate BODIPY FL vancomycin. We were puzzled by the potential roles of the two CwlM forms and investigated whether phosphorylation regulates the distribution of CwlM. We established that the phosphorylated CwlM form was mainly present in the cytoplasm of PknB-producing mycobacteria and was minimally detectable in the PknB-depleted *M. tuberculosis*. In contrast, the non-phosphorylated form was associated with the membrane; the T382A phosphoablative form of CwlM was found exclusively in the membrane and the phosphomimetic form was present predominantly in the cytoplasm of *cwlM*-CM. Our data suggest that the substitution of T382 with a negatively charged amino acid (T382D) does not fully mimic phosphorylated CwlM. Instead, CwlM T382D possessed properties of both the phosphorylated and non-phosphorylated forms, which explains how this CwlM form could complement the *cwlM*-CM but not the *pknB*-CM of *M. tuberculosis*. These results imply that a balance needs to be maintained between the phosphorylated and non-phosphorylated forms of CwlM and that this fine balance is essential for bacterial viability and can be affected by altered PknB expression or activity.

CwlM is predicted to be an *N*-acetylmuramoyl-L-alanine amidase; however, its actual activity remains uncertain. While Deng et al. (2005) have previously demonstrated CwlM to possess peptidoglycan hydrolyzing activity, in a more recent study, no such activity was detected, presumably due to the lack of two essential catalytic residues (Boutte et al., 2016). As

mentioned above, Boutte et al. (2016) have proposed that phospho-CwlM controls peptidoglycan generation by activating MurA; however, the possible functions of non-phosphorylated CwlM were not addressed. Previously published kinetic parameters do not support the activation of MurA by non-phosphorylated CwlM (Boutte et al., 2016). Moreover, *cwlM* could not be deleted in an *M. smegmatis* strain with an *murA* S368P mutation that rescued the growth defect of a phosphoablative *cwlM* mutant (Boutte et al., 2016).

In this study, we show that non-phosphorylated CwlM interacts with the essential linker region (E541–F680) of the proposed lipid II flippase, MurJ. Furthermore, phosphorylated CwlM does not interact with MurJ but instead binds to FhaA. CwlM-depleted mycobacteria did not incorporate BODIPY FL vancomycin and had a severe shape defect that can be attributed to impaired peptidoglycan biosynthesis (Figure S2). In mycobacteria, MurJ has an additional intracellular region that includes a pseudokinase domain (KHD) (Gee et al., 2012). KHD is phosphorylated by PknB to produce a complex with FhaA, but the precise role of this complex is not fully understood. Previously published data showed that the depletion of FhaA increased the incorporation of labeled vancomycin into peptidoglycan and that PknB overexpression had an opposite effect, increasing the accumulation of diaminopimelate (DAP)-containing precursors in the cytoplasm (Gee et al., 2012). It has been therefore proposed that PknB phosphorylation downregulates MurJ flippase activity (Gee et al., 2012). This potential regulatory mechanism is, however, non-essential for bacterial growth because FhaA and the pseudokinase domain of MurJ (D681–E955) could be inactivated without having any impact on mycobacterial viability (Gee et al., 2012).

We hypothesize that the binding of non-phosphorylated CwlM to the essential MurJ linker region is necessary for the function of MurJ, perhaps by facilitating the transport of lipid II across the membrane and activating peptidoglycan polymerization (Figure 6A). The nascent peptidoglycan is polymerized and incorporated into the existing cell wall during growth and cell division (Typas et al., 2011). This incorporation may be delayed under certain conditions, for example, when cell growth slows or when an efficient peptidoglycan synthesis complex causes peptidoglycan material to accumulate near the cell membrane, potentially interfering with other cell envelope processes. It was previously proposed that the PASTA domain of PknB senses uncrosslinked peptidoglycan (Yeats et al., 2002). The PASTA domain may thus bind such excessive peptidoglycan material (Figure 6B), resulting in the autophosphorylation and activation of PknB (Barthe et al., 2010), followed by the phosphorylation of CwlM and MurJ. In this scenario, the phosphorylated CwlM dissociates from MurJ and interacts with FhaA. Thus, PknB-mediated phosphorylation may control MurJ activity by two independent mechanisms: (1) by phosphorylating CwlM and preventing its interaction with MurJ and (2) by phosphorylating MurJ and inhibiting its activity. FhaA may serve as a regulatory hub to ensure that a balance is maintained between phosphorylated and non-phosphorylated CwlM and to regulate the interactions between CwlM and its partners, MurJ and MurA.

This PknB-mediated regulation perhaps supports the unique asymmetrical polar growth and peptidoglycan biosynthesis in mycobacteria (Joyce et al., 2012). Mycobacteria lack many important components to maintain cell shape, such as MreB (Hett and Rubin, 2008), and require properly matured peptidoglycan to preserve their rod-like shape and cell wall integrity (Baranowski et al., 2018).

Although the precise function and importance of the CwlM-MurJ interaction remain to be established, our data suggest that a distinct mechanism exists for the regulation of peptidoglycan synthesis in mycobacteria. The activation of MurJ by CwlM poses a significant technical challenge to demonstrate directly, because the possible flippase activity of MurJ has not been detected *in vitro*. CwlM may also be involved in the regulation of other cellular processes (e.g., via its interaction with CwsA) or it may possess other enzymatic activity. Future studies will thus help to establish the exact molecular mechanisms that underlie the essential role of CwlM in mycobacteria.

STAR★METHODS

Detailed methods are provided in the online version of this paper and include the following:

- KEY RESOURCES TABLE
- CONTACT FOR REAGENT AND RESOURCE SHARING
- EXPERIMENTAL MODEL AND SUBJECT DETAILS
- METHODS DETAILS
  - Generation of M. tuberculosis mutants
  - Peptidoglycan labeling and microscopy
  - Transcriptional Profiling
  - Mycobacterial protein fragment complementation assay
  - Mycobacterial cell fractionation
  - Isolation of recombinant proteins
  - Protein Electrophoresis and Western Blot
  - Immunoprecipitation assays
  - *In vitro* protein phosphorylation by PknB
  - Quantitative label-free phosphoproteomics analysis and phosphopeptide quantification
  - NMR chemical shift mapping
- QUANTIFICATION AND STATISTICAL ANALYSIS
- DATA AND SOFTWARE AVAILABILITY

SUPPLEMENTAL INFORMATION

Supplemental Information includes seven figures and five tables and can be found with this article online at https://doi.org/10.1016/j.celrep.2018.09.004.

ACKNOWLEDGMENTS

The following reagents were obtained through BEI Resources, NIAID, NIH: Monoclonal Anti-*M. tuberculosis* GlnA (Gene Rv2220), Clone IT-58 (CBA5) (produced *in vitro*), NR-13656; Monoclonal Anti-*Mycobacterium tuberculosis* GroEL2 (Gene Rv0440), Clone IT-70 (DCA4) (produced *in vitro*), NR-13657; Genomic DNA from *Mycobacterium tuberculosis*, Strain H37Rv, NR-48669. We acknowledge the Centre for Core Biotechnology Services at the University of Leicester for support with the containment level 3 experiments, the imaging of *M. tuberculosis*, and the analysis of mycobacterial proteins. We are grateful to Bandar Alrashid for the cloning of *cwsA* in pUAB400, Oliver Sampson for the optimization of $MurJ_{icd}$ expression, and Angélique DeVisch for assistance with the FhaA binding experiments. The project was supported by the UK

Biotechnology and Biological Sciences Research Council grants BB/H008586/1 and BB/P001513/1 (to G.V.M.), BB/P001289/1 (to W.V.), and Future Leaders Fellowship BB/N011945/1 (to P.J.M.); the French Infrastructure for Integrated Structural Biology (FRISBI) ANR-10-INBS-05 grant (to M.C.-G.); and the High Committee of Educational Development in Iraq (to B.K.).

AUTHOR CONTRIBUTIONS

Conceptualization, O.T., W.V., and G.V.M.; Methodology, A.S.-I., P.A., A.R.B., M.C.-G., and R.W.; Investigation, O.T., F.F., B.K., J.S., P.B., P.J.M., M.C.-G., and G.V.M.; Analysis, O.T., R.W., M.C.-G., A.R.B., P.A., and G.V.M.; Resources, F.F. and D.G.; Writing – Original Draft, G.V.M. and O.T.; Writing – Review & Editing, G.V.M., R.W., W.V., M.C.-G., P.M., and D.G.; Funding Acquisition, G.V.M., W.V., M.C.-G., P.M., and B.K.; Supervision, G.V.M., O.T., W.V., and M.C.-G.

DECLARATION OF INTERESTS

P.A. is a director and shareholder in Gemini Biosciences, Liverpool, UK. The other authors declare no competing interests.

Received: April 13, 2018
Revised: June 11, 2018
Accepted: August 31, 2018
Published: October 2, 2018

REFERENCES

Baek, S.H., Li, A.H., and Sassetti, C.M. (2011). Metabolic regulation of mycobacterial growth and antibiotic sensitivity. PLoS Biol. *9*, e1001065.

Baranowski, C., Lok-To, S., Eskandarian, H.A., Welsh, M.A., Lim, H.C., Kieser, K.J., Wagner, J.C., Walker, S., McKinney, J.D., Fantner, G.E., et al. (2018). Maturing mycobacterial peptidoglycan required non-canonical crosslinks to maintain shape. bioRxiv. https://doi.org/10.1101/291823.

Bartek, I.L., Woolhiser, L.K., Baughn, A.D., Basaraba, R.J., Jacobs, W.R., Jr., Lenaerts, A.J., and Voskuil, M.I. (2014). *Mycobacterium tuberculosis* Lsr2 is a global transcriptional regulator required for adaptation to changing oxygen levels and virulence. MBio *5*, e01106–e01114.

Barthe, P., Mukamolova, G.V., Roumestand, C., and Cohen-Gonsaud, M. (2010). The structure of PknB extracellular PASTA domain from *mycobacterium tuberculosis* suggests a ligand-dependent kinase activation. Structure *18*, 606–615.

Beilharz, K., Nováková, L., Fadda, D., Branny, P., Massidda, O., and Veening, J.W. (2012). Control of cell division in *Streptococcus pneumoniae* by the conserved Ser/Thr protein kinase StkP. Proc. Natl. Acad. Sci. USA *109*, E905–E913.

Bolla, J.R., Sauer, J.B., Wu, D., Mehmood, S., Allison, T.M., and Robinson, C.V. (2018). Direct observation of the influence of cardiolipin and antibiotics on lipid II binding to MurJ. Nat. Chem. *10*, 363–371.

Boutte, C.C., Baer, C.E., Papavinasasundaram, K., Liu, W., Chase, M.R., Meniche, X., Fortune, S.M., Sassetti, C.M., Ioerger, T.R., and Rubin, E.J. (2016). A cytoplasmic peptidoglycan amidase homologue controls mycobacterial cell wall synthesis. eLife *5*, e14590.

Canova, M.J., Veyron-Churlet, R., Zanella-Cleon, I., Cohen-Gonsaud, M., Cozzone, A.J., Becchi, M., Kremer, L., and Molle, V. (2008). The *Mycobacterium tuberculosis* serine/threonine kinase PknL phosphorylates Rv2175c: mass spectrometric profiling of the activation loop phosphorylation sites and their role in the recruitment of Rv2175c. Proteomics *8*, 521–533.

Carette, X., Platig, J., Young, D.C., Helmel, M., Young, A.T., Wang, Z., Potluri, L.P., Moody, C.S., Zeng, J., Prisic, S., et al. (2018). Multisystem analysis of *Mycobacterium tuberculosis* reveals kinase-dependent remodeling of the pathogen-environment interface. MBio *9*, e02333-17.

Chawla, Y., Upadhyay, S., Khan, S., Nagarajan, S.N., Forti, F., and Nandicoori, V.K. (2014). Protein kinase B (PknB) of *Mycobacterium tuberculosis* is essential for growth of the pathogen *in vitro* as well as for survival within the host. J. Biol. Chem. *289*, 13858–13875.

DeJesus, M.A., Gerrick, E.R., Xu, W., Park, S.W., Long, J.E., Boutte, C.C., Rubin, E.J., Schnappinger, D., Ehrt, S., Fortune, S.M., et al. (2017). Comprehensive essentiality analysis of the *Mycobacterium tuberculosis* genome via saturating transposon mutagenesis. MBio *8*, e02133-16.

Deng, L.L., Humphries, D.E., Arbeit, R.D., Carlton, L.E., Smole, S.C., and Carroll, J.D. (2005). Identification of a novel peptidoglycan hydrolase CwlM in *Mycobacterium tuberculosis*. Biochim. Biophys. Acta *1747*, 57–66.

Errington, J., Mickiewicz, K., Kawai, Y., and Wu, L.J. (2016). L-form bacteria, chronic diseases and the origins of life. Philos. Trans. R. Soc. Lond. B Biol. Sci. *371*, 20150494.

Fernandez, P., Saint-Joanis, B., Barilone, N., Jackson, M., Gicquel, B., Cole, S.T., and Alzari, P.M. (2006). The Ser/Thr protein kinase PknB is essential for sustaining mycobacterial growth. J. Bacteriol. *188*, 7778–7784.

Forti, F., Crosta, A., and Ghisotti, D. (2009). Pristinamycin-inducible gene regulation in mycobacteria. J. Biotechnol. *140*, 270–277.

Gee, C.L., Papavinasasundaram, K.G., Blair, S.R., Baer, C.E., Falick, A.M., King, D.S., Griffin, J.E., Venghatakrishnan, H., Zukauskas, A., Wei, J.R., et al. (2012). A phosphorylated pseudokinase complex controls cell wall synthesis in mycobacteria. Sci. Signal. *5*, ra7.

Hauck, S.M., Dietter, J., Kramer, R.L., Hofmaier, F., Zipplies, J.K., Amann, B., Feuchtinger, A., Deeg, C.A., and Ueffing, M. (2010). Deciphering membrane-associated molecular processes in target tissue of autoimmune uveitis by label-free quantitative mass spectrometry. Mol. Cell. Proteomics *9*, 2292–2305.

Haydel, S.E., Malhotra, V., Cornelison, G.L., and Clark-Curtiss, J.E. (2012). The *prrAB* two-component system is essential for *Mycobacterium tuberculosis* viability and is induced under nitrogen-limiting conditions. J. Bacteriol. *194*, 354–361.

Hett, E.C., and Rubin, E.J. (2008). Bacterial growth and cell division: a mycobacterial perspective. Microbiol. Mol. Biol. Rev. *72*, 126–156.

Joyce, G., Williams, K.J., Robb, M., Noens, E., Tizzano, B., Shahrezaei, V., and Robertson, B.D. (2012). Cell division site placement and asymmetric growth in mycobacteria. PLoS One *7*, e44582.

Kang, C.M., Abbott, D.W., Park, S.T., Dascher, C.C., Cantley, L.C., and Husson, R.N. (2005). The *Mycobacterium tuberculosis* serine/threonine kinases PknA and PknB: substrate identification and regulation of cell shape. Genes Dev. *19*, 1692–1704.

Kieser, K.J., Boutte, C.C., Kester, J.C., Baer, C.E., Barczak, A.K., Meniche, X., Chao, M.C., Rego, E.H., Sassetti, C.M., Fortune, S.M., and Rubin, E.J. (2015). Phosphorylation of the peptidoglycan synthase PonA1 governs the rate of polar elongation in mycobacteria. PLoS Pathog. *11*, e1005010.

Kumar, P., Arora, K., Lloyd, J.R., Lee, I.Y., Nair, V., Fischer, E., Boshoff, H.I., and Barry, C.E., 3rd. (2012). Meropenem inhibits D,D-carboxypeptidase activity in *Mycobacterium tuberculosis*. Mol. Microbiol. *86*, 367–381.

Lee, H., Yuan, C., Hammet, A., Mahajan, A., Chen, E.S., Wu, M.R., Su, M.I., Heierhorst, J., and Tsai, M.D. (2008). Diphosphothreonine-specific interaction between an SQ/TQ cluster and an FHA domain in the Rad53-Dun1 kinase cascade. Mol. Cell *30*, 767–778.

Leiba, J., Carrère-Kremer, S., Blondiaux, N., Dimala, M.M., Wohlkönig, A., Baulard, A., Kremer, L., and Molle, V. (2014). The *Mycobacterium tuberculosis* transcriptional repressor EthR is negatively regulated by serine/threonine phosphorylation. Biochem. Biophys. Res. Commun. *446*, 1132–1138.

Mir, M., Asong, J., Li, X., Cardot, J., Boons, G.J., and Husson, R.N. (2011). The extracytoplasmic domain of the *Mycobacterium tuberculosis* Ser/Thr kinase PknB binds specific muropeptides and is required for PknB localization. PLoS Pathog. *7*, e1002182.

Mohammadi, T., van Dam, V., Sijbrandi, R., Vernet, T., Zapun, A., Bouhss, A., Diepeveen-de Bruin, M., Nguyen-Distèche, M., de Kruijff, B., and Breukink, E. (2011). Identification of FtsW as a transporter of lipid-linked cell wall precursors across the membrane. EMBO J. *30*, 1425–1432.

Molle, V., Brown, A.K., Besra, G.S., Cozzone, A.J., and Kremer, L. (2006). The condensing activities of the *Mycobacterium tuberculosis* type II fatty acid

synthase are differentially regulated by phosphorylation. J. Biol. Chem. *281*, 30094–30103.

Munshi, T., Gupta, A., Evangelopoulos, D., Guzman, J.D., Gibbons, S., Keep, N.H., and Bhakta, S. (2013). Characterisation of ATP-dependent Mur ligases involved in the biogenesis of cell wall peptidoglycan in *Mycobacterium tuberculosis*. PLoS One *8*, e60143.

Nagarajan, S.N., Upadhyay, S., Chawla, Y., Khan, S., Naz, S., Subramanian, J., Gandotra, S., and Nandicoori, V.K. (2015). Protein kinase A (PknA) of *Mycobacterium tuberculosis* is independently activated and is critical for growth *in vitro* and survival of the pathogen in the host. J. Biol. Chem. *290*, 9626–9645.

Parikh, A., Verma, S.K., Khan, S., Prakash, B., and Nandicoori, V.K. (2009). PknB-mediated phosphorylation of a novel substrate, N-acetylglucosamine-1-phosphate uridyltransferase, modulates its acetyltransferase activity. J. Mol. Biol. *386*, 451–464.

Pensinger, D.A., Schaenzer, A.J., and Sauer, J.D. (2018). Do shoot the messenger: PASTA kinases as virulence determinants and antibiotic targets. Trends Microbiol. *26*, 56–69.

Pereira, S.F., Goss, L., and Dworkin, J. (2011). Eukaryote-like serine/threonine kinases and phosphatases in bacteria. Microbiol. Mol. Biol. Rev. *75*, 192–212.

Prigozhin, D.M., Papavinasasundaram, K.G., Baer, C.E., Murphy, K.C., Moskaleva, A., Chen, T.Y., Alber, T., and Sassetti, C.M. (2016). Structural and genetic analyses of the *Mycobacterium tuberculosis* protein kinase B sensor domain identify a potential ligand-binding site. J. Biol. Chem. *291*, 22961–22969.

Prisic, S., and Husson, R.N. (2014). *Mycobacterium tuberculosis* serine/threonine protein kinases. Microbiol. Spectr. *2*, Published online October, 2014. https://doi.org/10.1128/microbiolspec.MGM2-0006-2013.

Prisic, S., Dankwa, S., Schwartz, D., Chou, M.F., Locasale, J.W., Kang, C.M., Bemis, G., Church, G.M., Steen, H., and Husson, R.N. (2010). Extensive phosphorylation with overlapping specificity by *Mycobacterium tuberculosis* serine/threonine protein kinases. Proc. Natl. Acad. Sci. USA *107*, 7521–7526.

Rossi, F., Khanduja, J.S., Bortoluzzi, A., Houghton, J., Sander, P., Güthlein, C., Davis, E.O., Springer, B., Böttger, E.C., Relini, A., et al. (2011). The biological and structural characterization of *Mycobacterium tuberculosis* UvrA provides novel insights into its mechanism of action. Nucleic Acids Res. *39*, 7316–7328.

Roumestand, C., Leiba, J., Galophe, N., Margeat, E., Padilla, A., Bessin, Y., Barthe, P., Molle, V., and Cohen-Gonsaud, M. (2011). Structural insight into the *Mycobacterium tuberculosis* Rv0020c protein and its interaction with the PknB kinase. Structure *19*, 1525–1534.

Shah, I.M., Laaberki, M.H., Popham, D.L., and Dworkin, J. (2008). A eukaryotic-like Ser/Thr kinase signals bacteria to exit dormancy in response to peptidoglycan fragments. Cell *135*, 486–496.

Sham, L.T., Butler, E.K., Lebar, M.D., Kahne, D., Bernhardt, T.G., and Ruiz, N. (2014). Bacterial cell wall. MurJ is the flippase of lipid-linked precursors for peptidoglycan biogenesis. Science *345*, 220–222.

Singh, A., Mai, D., Kumar, A., and Steyn, A.J. (2006). Dissecting virulence pathways of *Mycobacterium tuberculosis* through protein-protein association. Proc. Natl. Acad. Sci. USA *103*, 11346–11351.

Squeglia, F., Romano, M., Ruggiero, A., and Berisio, R. (2017). Molecular players in tuberculosis drug development: another break in the cell wall. Curr. Med. Chem. *24*, 3954–3969.

Thingholm, T.E., Jørgensen, T.J., Jensen, O.N., and Larsen, M.R. (2006). Highly selective enrichment of phosphorylated peptides using titanium dioxide. Nat. Protoc. *1*, 1929–1935.

Turapov, O., Loraine, J., Jenkins, C.H., Barthe, P., McFeely, D., Forti, F., Ghisotti, D., Hesek, D., Lee, M., Bottrill, A.R., et al. (2015). The external PASTA domain of the essential serine/threonine protein kinase PknB regulates mycobacterial growth. Open Biol. *5*, 150025.

Typas, A., Banzhaf, M., Gross, C.A., and Vollmer, W. (2011). From the regulation of peptidoglycan synthesis to bacterial growth and morphology. Nat. Rev. Microbiol. *10*, 123–136.

Villarino, A., Duran, R., Wehenkel, A., Fernandez, P., England, P., Brodin, P., Cole, S.T., Zimny-Arndt, U., Jungblut, P.R., Cerveñansky, C., and Alzari, P.M. (2005). Proteomic identification of *M. tuberculosis* protein kinase substrates: PknB recruits GarA, a FHA domain-containing protein, through activation loop-mediated interactions. J. Mol. Biol. *350*, 953–963.

Vornhagen, J., Burnside, K., Whidbey, C., Berry, J., Qin, X., and Rajagopal, L. (2015). Kinase inhibitors that increase the sensitivity of methicillin resistant Staphylococcus aureus to β-lactam antibiotics. Pathogens *4*, 708–721.

Wayne, L.G., and Sohaskey, C.D. (2001). Nonreplicating persistence of *mycobacterium tuberculosis*. Annu. Rev. Microbiol. *55*, 139–163.

Wiśniewski, J.R., Zougman, A., Nagaraj, N., and Mann, M. (2009). Universal sample preparation method for proteome analysis. Nat. Methods *6*, 359–362.

World Health Organization (2017). Tuberculosis (TB). Global tuberculosis report 2017. http://www.who.int/tb/publications/global_report/en/.

Wu, F.L., Liu, Y., Jiang, H.W., Luan, Y.Z., Zhang, H.N., He, X., Xu, Z.W., Hou, J.L., Ji, L.Y., Xie, Z., et al. (2017). The Ser/Thr protein kinase protein-protein interaction map of *M. tuberculosis*. Mol. Cell. Proteomics *16*, 1491–1506.

Yeats, C., Finn, R.D., and Bateman, A. (2002). The PASTA domain: a beta-lactam-binding domain. Trends Biochem. Sci. *27*, 438.

Zahrt, T.C., and Deretic, V. (2000). An essential two-component signal transduction system in *Mycobacterium tuberculosis*. J. Bacteriol. *182*, 3832–3838.

Zhang, M., Chen, J.M., Sala, C., Rybniker, J., Dhar, N., and Cole, S.T. (2014). EspI regulates the ESX-1 secretion system in response to ATP levels in *Mycobacterium tuberculosis*. Mol. Microbiol. *93*, 1057–1065.

Zucchini, L., Mercy, C., Garcia, P.S., Cluzel, C., Gueguen-Chaignon, V., Galisson, F., Freton, C., Guiral, S., Brochier-Armanet, C., Gouet, P., and Grangeasse, C. (2018). PASTA repeats of the protein kinase StkP interconnect cell constriction and separation of *Streptococcus pneumoniae*. Nat. Microbiol. *3*, 197–209.

STAR★METHODS

KEY RESOURCES TABLE

REAGENT or RESOURCE	SOURCE	IDENTIFIER
Antibodies		
Phospho-Threonine Antibody (P-Thr Polyclonal)	Cell Signaling Technology	Cat#9381, RRID:AB_330301
Monoclonal Anti-polyHistidine antibody produced in mouse	Sigma-Alrdich	Cat#H1029, RRID:AB_260015
Monoclonal Anti-*M. tuberculosis* GlnA (Gene Rv2220), Clone IT-58	BEI Resources	NR-13656
Monoclonal Anti-*M. tuberculosis* GroEL2 (Gene Rv0440), Clone IT-70	BEI Resources	NR-13657
Anti-PknB antibody raised in rabbit	Forti et al., 2009	N/A
Custom anti-GarA antibody raised in rabbit	Cambridge Biosciences provided by H O'Hare	N/A
Custom polyclonal anti-CwlM antibody raised in rabbit	Thermo Fisher Scientific	N/A
Custom polyclonal antibodies raised against GKNDRPT-phosphoGT in rabbit (anti-T382-P)	Gemini Biosciences Ltd	N/A
Custom polyclonal antibody raised against GKNDRPTGT in rabbit (anti-T382)	Gemini Biosciences Ltd	N/A
Anti-Mouse IgG (whole molecule) –Alkaline Phosphatase antibody produced in rabbit	Sigma-Aldrich	Cat# A3562; RRID:AB_258091
Mouse Anti-Rabbit IgG Antibody: AP	Aviva Systems Biology via Generon	Cat# OASB00822
Anti-rabbit IgG, HRP-linked Antibody	Cell Signaling Technology	Cat#7074; RRID:AB_2099233
Bacterial and Virus Strains		
Mycobacterium tuberculosis H37Rv	Laboratory stock	ATCC 27294
Mycobacterium smegmatis mc^{2}155	Laboratory stock	ATCC 700084
Mycobacterium tuberculosis H37Rv conditional PknB mutant	Forti et al., 2009	N/A
Mycobacterium tuberculosis H37Rv conditional CwlM mutant	This study	N/A
Mycobacterium tuberculosis H37Rv conditional CwlM complemented mutants (detailed in Table S2)	This study	N/A
Mycobacterium smegmatis mc^{2}155 M-PFC strains (detailed in Table S2)	This study	N/A
Escherichia coli α-Select Gold Competent Cells	BIOLINE	BIO-85027
Escherichia coli OverExpress C41 (DE3) Chemically Competent Cells	Lucigen	Cat#60442-1
Escherichia coli OverExpress C41 (DE3) strains for overexpression of recombinant proteins (detailed in Table S2)	This study	N/A
Chemicals, Peptides, and Recombinant Proteins		
BD Difco Dehydrated Culture Media: Middlebrook 7H9 Broth	Fisher Scientific	*Cat#DF0713-17-9*
BD Difco Dehydrated Culture Media: Middlebrook 7H10 Agar	Fisher Scientific	*Cat#DF0627-17-4*
Hygromycin B (50 mg/ml)	ThermoFisher Scientific	Cat#10687010
Pristinamycin	Molcan	Cat# PSM01A-100
cOmplete Ultra Tablets Protease Inhibitor Cocktail	Sigma-Aldrich	*05892970001 ROCHE*
PhosSTOP phosphatase inhibitor tablets	Sigma-Aldrich	*PHOSSRO ROCHE*
Ni-NTA agarose	QIAGEN	Cat#30210
Glutathione Sepharose 4B GST-tagged protein purification resin	GE Healthcare Life Sciences	Cat#17075601
HiLoad 16/600 Superdex 200 pg prepacked column	GE Healthcare Life Sciences	Cat#28989335
Cyanogen bromide-activated-Sepharose 4B	Sigma-Aldrich	Cat#C9210
AC-NDRPTGTFTFAELLA-NH2 peptide	Generon	Custom synthesized

(*Continued on next page*)

Continued

REAGENT or RESOURCE	SOURCE	IDENTIFIER
AC-NDRPT(phospo)GTFT(phospo)FAELLA-NH2	Generon	Custom synthesized
AC-NDRPT(phospho)GTFTFAELLA-NH2	Generon	Custom synthesized
AC-NDRPTGTFT(phospho)FAELLA-NH2	Generon	Custom synthesized
AC-NDRPAGTFAFAELLA-NH2	Generon	Custom synthesized
AC-NDRPDGTFDFAELLA-NH2	Generon	Custom synthesized
SERVA Gel TG Prime 4-20% 10 samples wells	Generon	Cat# 43277.01
SERVA Gel TG Prime 12% 12 samples wells	Generon	Cat# 43266.01
SIGMAFAST BCIP/NBT	Sigma-Aldrich	Cat#5655
BODIPY FL Vancomycin	ThermoFisher Scientific	*Cat# V34850*
Trizol LS Reagent	ThermoFisher Scientific	*Cat#10296010*
Recombinant 6xHis-tagged CwlM	This study	*N/A*
Recombinant 6XHis-tagged MurJ ICD	This study	*N/A*
Recombinant 6xHis-tagged 6xHis-MurJ$_{E541-F680}$	This study	*N/A*
Recombinant GST-tagged FhaA	This study	*N/A*
Rv0020c_FHA domain	Roumestand et al., 2011	*N/A*
Recombinant GST-tagged Wag31	This study	*N/A*
Critical Commercial Assays		
GenElute Plasmid Miniprep kit	Sigma-Aldrich	*Cat# PLN350*
QIAquick Gel Extraction Kit	QIAGEN	Cat# 28706
QIAquick PCR Purification Kit	QIAGEN	Cat# 28106
GeneArt Site-Directed Mutagenesis PLUS System	ThermoFisher Scientific	*Cat#A14604*
Turbo DNA-free kit	ThermoFisher Scientific	*Cat#AM1907*
SuperScript Reverse Transcriptase II	ThermoFisher Scientific	*Cat#18064022*
Absolute QPCR SYBR Green mix	ThermoFisher Scientific	*Cat#AB4322B*
Platinum *Taq* DNA polymerase	ThermoFisher Scientific	*Cat# 10966034*
Restriction enzymes	New England Biolabs(UK) Ltd- /	Cat #R3193S; R3182L; R0111L; R3136L; R3142L
LigaFast Rapid DNA Ligation System	Promega	Cat# M8221
SignalFire Elite ECL Reagent	Cell Signaling Technology	Cat#12757S
Titansphere Phos-TiO Kit	GL Sciences	Cat# 5010-21311
Deposited Data		
Raw and analyzed phosphoproteomics data	ProteomeXchange Consortium via the PRIDE	PXD009239 and 10.6019/PXD009239
Oligonucleotides		
Oligonucleotides were custom synthesized (details provided in Table S4)	Sigma Aldrich	N/A
Recombinant DNA		
Purified Genomic DNA from NR-13648 *M. tuberculosis* Strain H37Rv	BEI-Resources	NR-48669
Mycobacterial protein fragment complementation (M-PFC)	Singh et al., 2006	N/A
Integrating plasmid pMV306	Baek et al., 2011	N/A
pAZI9479 suicide vector.	Forti et al., 2009	N/A
pET15bTEV	Canova et al., 2008	N/A
GST-tagged PknB-(1-331)	Molle et al., 2006	N/A
Software and Algorithms		
Xcalibur software	ThermoFisher Scientific	Version 2.0 SR2 Core, RRID:SCR_014593
Progenesis LC-MS software	Nonlinear Dynamics Hauck et al., 2010	Version 2.4, Nonlinear

(*Continued on next page*)

Continued

REAGENT or RESOURCE	SOURCE	IDENTIFIER
MASCOT	Matrix Science, London, UK	Version 2.2.04, RRID:SCR_014322
Scaffold Q+	Proteome Software Inc., Portland, OR	Version 4.8.1
Proteome Discoverer	Thermo Scientific	Version 1.4.1.14, RRID:SCR_014477
X!Tandem	The GPM, thegpm.org	Version CYCLONE 2010.12.01.1, RRID:SCR_015645

CONTACT FOR REAGENT AND RESOURCE SHARING

Further information and requests for reagents should be directed to and will be fulfilled by the Lead Contact, Galina V. Mukamolova (gvm4@leicester.ac.uk).

EXPERIMENTAL MODEL AND SUBJECT DETAILS

M. tuberculosis and *M. smegmatis* were grown in Middlebrook 7H9 liquid medium supplemented with 10% (v/v) Albumin-Dextrose Complex (ADC), 0.2% (v/v) glycerol and 0.1% (w/v) at 37°C with shaking at 100 rpm. Antimicrobials were added at the following concentrations (μg/ml): hygromycin 50; kanamycin 50; pristinamycin 0.5; trimethoprim 15. Sucrose magnesium medium (SMM) contained 0.3 M sucrose, 20 mM $MgSO_4$, 0.1% Tween 80 (w/v), 10% (v/v) ADC in standard 7H9 broth. Bacterial growth was followed by measurement of absorbance at 580 nm, using a spectrophotometer, or by colony-forming unit (CFU) counting on 7H10 agar.

METHODS DETAILS

Generation of M. tuberculosis mutants

To generate *cwlM*-CM, a 5′-prime fragment of the *cwlM* gene (800 bp) from *M. tuberculosis* was amplified using primers CMRv3915F and CMRv3915R for *M. tuberculosis* (Table S5). This fragment was cloned into *NcoI* and *SphI* sites of the pAZI9479 plasmid. Transformants were selected on 7H10 agar containing hygromycin and pristinamycin. Single crossovers were confirmed by PCR using primers FG2224 and FG3106.

For *cwlM*-CM complementation, a coding sequence of *Rv3915* (*cwlM*) with a 200 bp-upstream region was amplified from the *M. tuberculosis* genome using primers Rv3915pMV306F2 and Rv3915pMV306R1. The resulting fragment was cloned into the KpnI and HindIII sites of the pMV306 plasmid. Transformants were selected on 7H10 medium containing hygromycin, kanamycin and pristinamycin. CwlM variants were obtained using a GeneArt Site-Directed Mutagenesis System and the primers used are listed in Table S5. All constructs were sequenced by GATC Biotech before further applications.

Peptidoglycan labeling and microscopy

M. tuberculosis cells were incubated with a mixture of vancomycin and BODIPY FL vancomycin for 24 hours with shaking at 37°C. Mycobacteria were washed with PBS and fixed in 2% (w/v) paraformaldehyde in PBS for 24 hours before imaging using a 12/10bit, high-speed Peltier-cooled CCD camera (FDI, Photonic Science) using Image-Pro Plus (Media Cybernetics) software.

For scanning electron microscopy (SEM), mycobacteria from exponential phase were washed in PBS before fixation in 2.5% glutaraldehyde in PBS for 24 hours at room temperature. After further PBS washes, cells were dispensed onto a poly-L-lysine coated glass slide, before further fixation with 1% aqueous osmium tetroxide at room temperature. Extensively washed glass slides were mounted onto aluminum stubs, coated with gold/palladium in a Quorum Q150 TES coating unit, and were then imaged using a Hitachi S3000H SEM with an accelerating voltage of 10kV.

Transcriptional Profiling

Total RNA was isolated from 10 mL of mycobacterial cultures using the Trizol reagent, and cDNA samples were generated using Superscript Reverse Transcriptase II and gene-specific primers. Q-PCR was performed in a Corbett Rotor Gene 6000 real time thermocycler using Absolute QPCR SYBR Green mix, as described previously (Turapov et al., 2015).

Mycobacterial protein fragment complementation assay

Genes of interest were amplified from the *M. tuberculosis* genome and were cloned in corresponding plasmids. *CwlM* was cloned in pUAB100 (replacing the GCN4 leucine zipper domain) and in pUAB300 (Singh et al., 2006) to generate fusion proteins with dihydrofolate reductase domains. Full length *dnaA, fhaA, ftsE, ftsZ*, *cwsA* and *murJicd* were cloned in pUAB200 (replacing the GCN4 leucine zipper domain) and pUAB400 plasmids. *M. smegmatis* transformants were spotted on 7H10 plates supplemented with hygromycin, kanamycin and trimethoprim.

Mycobacterial cell fractionation

Mycobacteria were lysed in a Minilys homogenizer (Bertin Instruments) using glass beads in TBS buffer containing 20 mM TrisCl, pH 8.0, 150 mM NaCl, 20 mM KCl, 10 mM $MgCl_2$, and proteinase/phosphatase inhibitors. Lysates were centrifuged at 27,000 x *g* for 1 hour (pellets discarded), followed by 4-hour centrifugation at 100,000 x *g*. The supernatants contained cytoplasmic proteins (cytoplasmic fraction); the pellets (membrane fractions) were washed once in carbonate buffer, pH 11 and twice in TBS buffer. Proteins from cellular fractions were separated on SDS-PAGE. The purity of fractions was confirmed by the detection of diagnostic proteins, GarA (cytoplasmic protein) and GlnA (membrane protein).

Isolation of recombinant proteins

CwlM, *murJicd, murJ*$_{E541\text{-}F680}$, *fhaA, wag31* were amplified from the *M. tuberculosis* genome using corresponding primers (Table S2) and were cloned either in pET15-TEV (*cwlM*, *murJcd, murJ*$_{E541\text{-}F680}$) or in pGEX2T (*fhaA* and *wag31*). After confirmation by sequencing, the constructs were transformed into *E. coli* OverExpress C41(DE3) competent cells. *E. coli* strains were grown to OD 0.5 and protein expression was induced with 0.5 mM IPTG followed by incubation at 16°C overnight. The recombinant proteins were purified using affinity chromatography and size exclusion chromatography.

Protein Electrophoresis and Western Blot

Proteins were separated on 4%–20% gradient SERVA gels and transferred onto a nitrocellulose membrane using a Trans-Blot® Turbo Transfer System (Bio-Rad). SIGMAFAST BCIP®/NBT or SignalFire Elite ECL Reagent were used to visualize proteins on C-DiGit Chemiluminescent Blot Scanner (LI-COR Biosciences), according to the manufacturer's instructions.

Immunoprecipitation assays

Anti-CwlM-IgG Sepharose was prepared by cross-linking the anti-CwlM antibody to cyanogen bromide-activated-Sepharose® 4B. For immunoprecipitation assays, cellular fractions (100 μg proteins in 1mL) were mixed with 10 μL of anti-CwlM-IgG-Sepharose and incubated for 60 min on a laboratory rotator, followed by centrifugation for 5 min at 500 x g. Supernatants were removed and the resin pellets were washed 3 times with TBS. Proteins were extracted with 40 μL of phosphoric acid, pH 2.0, dried and used for western blot and mass spectrometry analyses. Cellular fractions from the CwlM-depleted mutant served as a control to detect non-specifically binding or contaminating proteins.

For confirmation of interactions, recombinant CwlM and other proteins (10 μg each) were mixed in phosphate buffer (20 mM KH_2PO_4, pH 7.0, 100 mM NaCl, 10 mM KCl) with 10 μL of anti-CwlM-IgG-Sepharose and processed as described above.

In vitro protein phosphorylation by PknB

Purified recombinant CwlM (10 μM) was mixed with the recombinant catalytic domain of PknB (5 μM) in a kinase buffer (20 mM Tris–HCl, pH 8.0; 0.5 mM DTT; 10 mM $MgCl_2$; 0.1 mM ATP) and incubated at 37°C for one hour. To identify phosphorylated residues, trypsin-digested proteins were analyzed using a LTQ-Orbitrap-Velos mass spectrometer.

Quantitative label-free phosphoproteomics analysis and phosphopeptide quantification

PknB-CM cultures were centrifuged, washed twice in PBS and resuspended in buffer containing 20 mM TrisCl, pH 7.5, 1 M NaCl, 8 M urea, and proteinase/phosphatase inhibitors. After bead beating, lysates were cleared by centrifugation and filtration (0.22 μm) and treated using the FASP protocol, as described previously (Wiśniewski et al., 2009). Desalted samples were enriched on TiO_2 beads (Thingholm et al., 2006), speed vacuumed to dryness and re-suspended in 1% formic acid. Trypsin-digested peptides were separated using an Ultimate 3000 RSLC (Thermo Scientific) nanoflow LC system and Acclaim PepMap100 nanoViper C18 trap column (100 μm inner-diameter, 2cm; Thermo Scientific). After trap enrichment, peptides were eluted onto an Acclaim PepMap RSLC nanoViper, C18 column (75 μm, 15 cm; ThermoScientific) with a linear gradient of 2%–40% solvent B (80% acetonitrile with 0.08% formic acid). The HPLC system was coupled to a linear ion trap Orbitrap hybrid mass spectrometer (LTQ-Orbitrap Velos, Thermo Scientific) via a nanoelectrospray ion source (Thermo Scientific). Data were acquired using the Xcalibur software. The acquired spectra (Thermo.raw files) were loaded to the Progenesis LC-MS software (version 2.4, Nonlinear) for label free quantification (Hauck et al., 2010). Three biological replicates for each sample were analyzed. Profile data of the MS scans were transformed to peak lists with Progenesis LC-MS using a proprietary algorithm. The database search was performed with MASCOT (version 2.3.2, Matrix Science, London, UK).

NMR chemical shift mapping

FHA ^1H_N and ^{15}N backbone chemical shift perturbations (Δδ) were measured from ^1H-^{15}N HSQC experiments upon titration with different peptides corresponding to the C-terminal phosphorylated part of CwlM (^{378}NDRPTGTFTFAELLA^{392}). ^1H-^{15}N HSQC experiments were carried out at 20°C on a Bruker Avance III 800 spectrometer, equipped with 5 mm z-gradient TCI cryoprobe. ^{15}N-labeled Rv0020c-FHA domain (80 μM) was dissolved in 10 mM sodium phosphate buffer, pH 6.8, 100 mM NaCl, 1 mM Tris-HCl with 5% D_2O for the lock. Six spectra were recorded by adding 80 μM of six different peptides corresponding to different phosphorylation (p) states of the C-terminal part of CwlM: pT382-T386, T382-pT386, pT382-pT386, T382-T386, A382-A386 and D382-D386. An additional reference spectrum was taken on a FHA sample without peptides. All ^1H -^{15}N HSQC spectra were recorded using a time domain

data size of 64 (*t1*) × 1024 (*t2*) complex points, and 16 transients per *t1* increment. For analysis, ^1H_N and ^{15}N chemical shift changes were combined using the equation: $\Delta\delta = [(\Delta\delta_H)^2 + (\Delta\delta_N \times (\gamma_N/\gamma_H))^2]^{0.5}$, where values of $\Delta\delta > 0.078$ ppm have been defined as sign.

QUANTIFICATION AND STATISTICAL ANALYSIS

Analysis of growth (Figures 1, 2, S2) was done using Microsoft Excel for Mac Version 15.40. N correspond to independent biological replicates.

Quantitative label-free phosphoproteomics analysis and phosphopeptide quantification (Table 1 and Table S1): data were acquired using the Xcalibur software and acquired spectra (Thermo.raw files) were loaded to the Progenesis LC-MS software (version 2.4, Nonlinear) for label free quantification (Hauck et al., 2010). Three biological replicates for each sample were analyzed. Profile data of the MS scans were transformed to peak lists with Progenesis LC-MS using a proprietary algorithm. The database search was performed with MASCOT (version 2.3.2, Matrix Science, London, UK).

Densitometric analyses of protein bands (Figures 1 and 4) were done using ImageJ version 1.51 software. Blots or gels from three independent experiments were used. PknB intensity was expressed as percentage of PknB produced in the presence of pristinamycin which corresponds to band 2. Protein abundance bound to anti-CwlM IgG Sepharose was expressed as a percentage of total amount used for immunoprecipitation assays, which corresponds to lane 5 on each gel.

DATA AND SOFTWARE AVAILABILITY

Genebank: ASM19595v2 was used for annotation of *M. tuberculosis* proteins (https://www.ncbi.nlm.nih.gov/assembly/GCF_000195955.2/).

The accession numbers for the mass spectrometry proteomics data reported in this paper are ProteomeXchange Consortium via PRIDE: PXD009239 and 10.6019/PXD009239 (http://www.proteomexchange.org).

Cell Reports, Volume *25*

Supplemental Information

Two Faces of CwlM, an Essential PknB Substrate, in *Mycobacterium tuberculosis*

Obolbek Turapov, Francesca Forti, Baleegh Kadhim, Daniela Ghisotti, Jad Sassine, Anna Straatman-Iwanowska, Andrew R. Bottrill, Patrick J. Moynihan, Russell Wallis, Philippe Barthe, Martin Cohen-Gonsaud, Paul Ajuh, Waldemar Vollmer, and Galina V. Mukamolova

SUPPLEMENTAL FIGURES

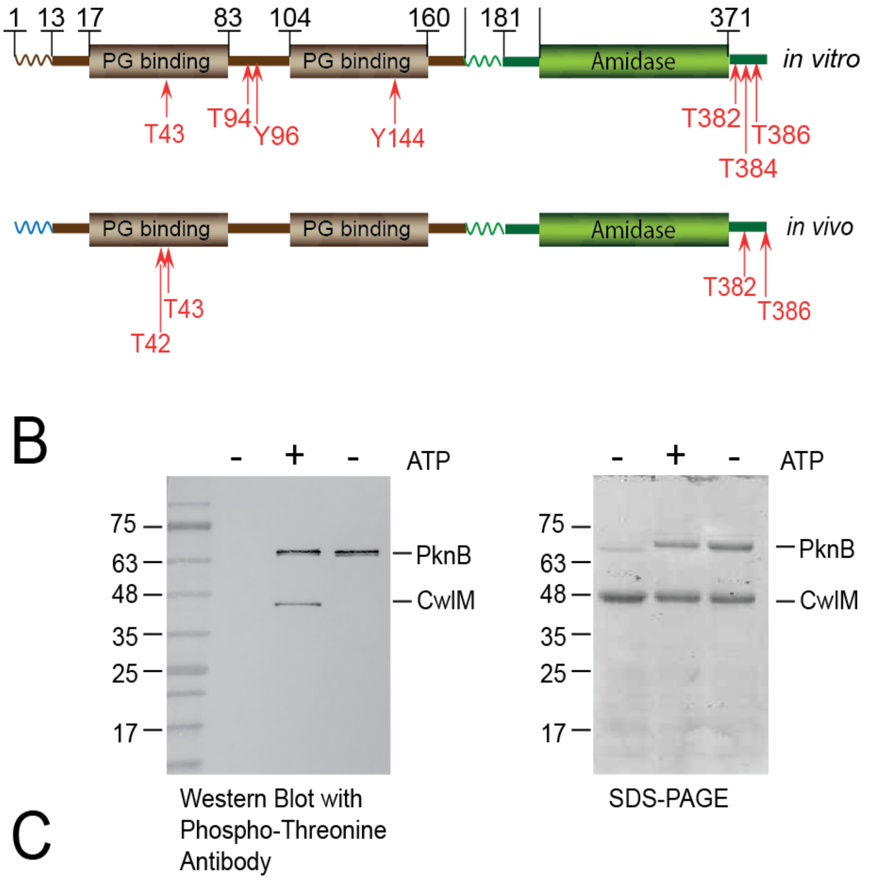

C

Peptide	Phospho residue	Observed mass (charge)	Actual mass
AALTALGMLDHQEEDLTTGR	T43	1111.52 (2)	2221.03
RLGARTLYHQFGAPLYGDDVATLQAR	T94	938.47 (3)	2814.4
RLGARTLYHQFGAPLYGDDVATLQAR	Y96	704.11 (4)	2812.4
EYGLAADGICGPETLR	Y144	595.93 (3)	1784.78
NDRPTGTFTFAELLAHELSVER	T382	862.08 (3)<br>646.82 (4)	2583.22<br>2583.22
NDRPTGTFTFAELLAHELSVER	T384	646.81 (4)<br>862.09 (4)	2583.22
NDRPTGTFTFAELLAHELSVER	T382<br>T384	888.74 (3)	2663.2
NDRPTGTFTFAELLAHELSVER	T382<br>T386	888.41 (3)	2662.2
NDRPTGTFTFAELLAHELSVER	T386	861.76 (3)	2582.26
NDRPTGTFTFAELLAHELSVER	T384<br>T386	888.41 (3)	2662.20

Figure S1. Phosphorylation of CwlM by PknB *in vitro*. Related to Table 1. (A) Schematic representation of phosphosites detected *in vitro* (top) and in the phosphoproteomics study (bottom). (B) A recombinant CwlM was phosphorylated by the recombinant enzymatic domain of PknB. Detection of phosphorylated proteins by Western blot analysis using phospho-threoinine antibody. (C) Phosphopeptides detected by mass-spectrometry.

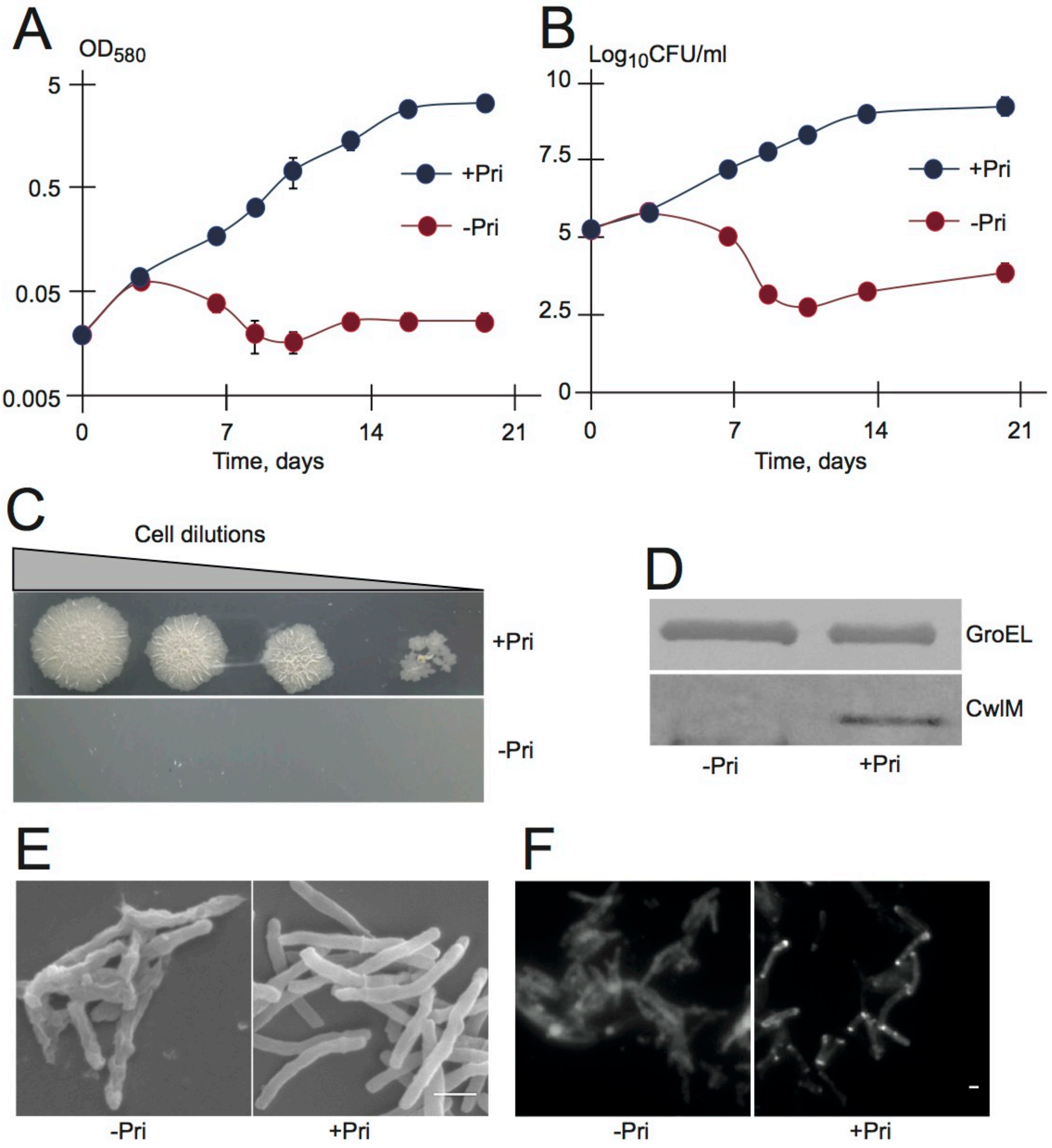

Figure S2. CwlM is essential for growth of *M. tuberculosis.* Related to Figure 2. See also Figure S3 and Table S3. A conditional *cwlM* mutant was grown in 7H9 medium with and without pristinamycin (Pri) at 37°C with shaking. Growth was monitored by measurement of OD at 580 nm (A) or by CFU counting on 7H10 agar (B). Data are represented as mean±SEM. (C) Growth of *cwlM*-CM on agar. (D) Detection of CwlM using western blot with anti-CwlM antibody. (E) Scanning electron micrographs of CwlM-producing and CwlM-depleted mycobacteria grown in standard 7H9 medium. (F) Detection of nascent peptidoglycan by Van-BODIPY labelling, data shown for mycobacteria grown in 7H9 medium, similar results were obtained in SMM (not shown for clarity). Scale bars – 1 μm.

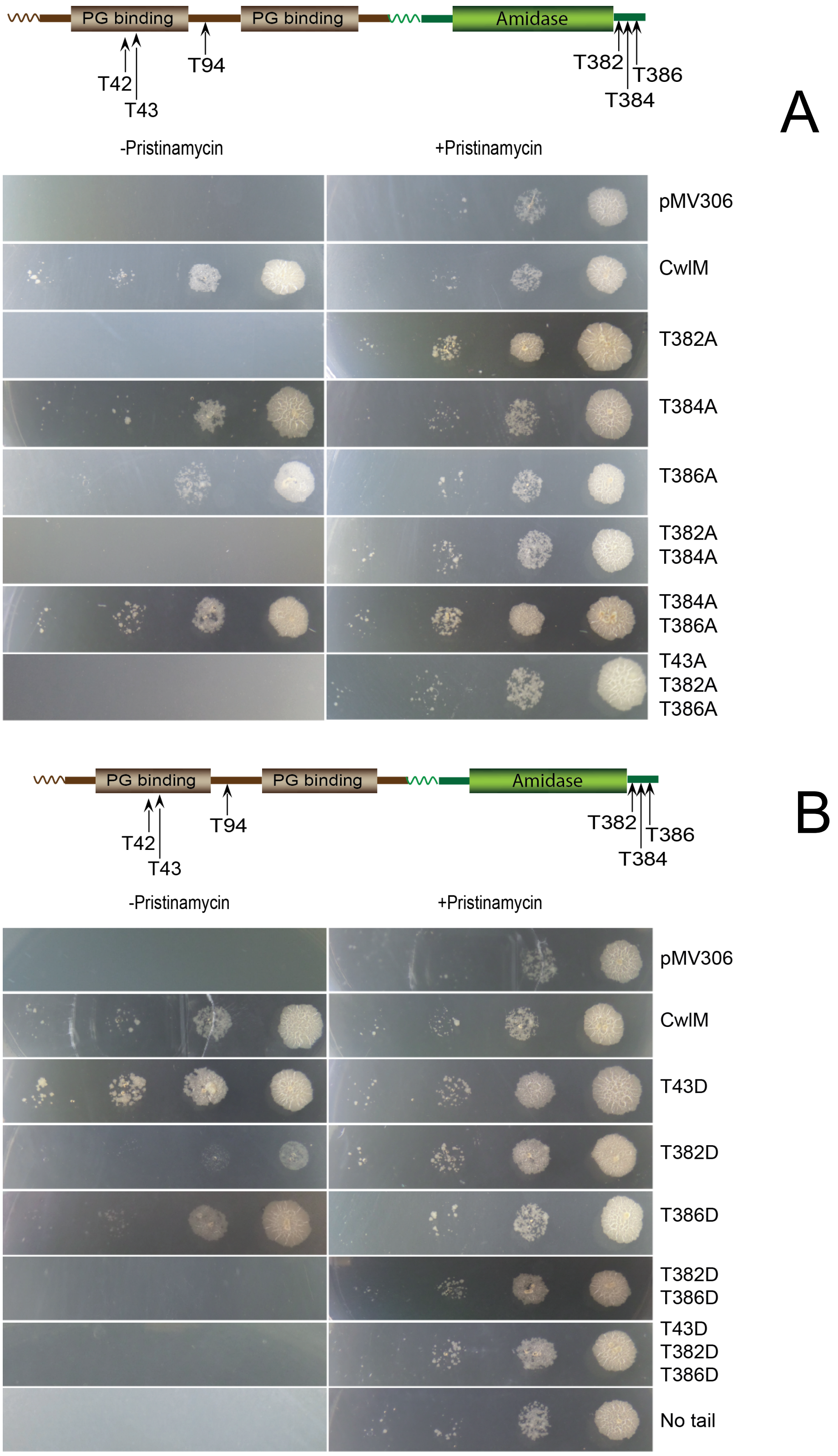

Figure S3. Growth of phosphoablative (A) and phosphomimetic (B) *M. tuberculosis* CwlM mutants on 7H10 agar. Related to Figure 2. See also Table S3. Serially diluted mycobacteria were plated on 7H10 agar with kanamycin and hygromycin +/-pristinamycin and incubated for 4 weeks at 37°C.

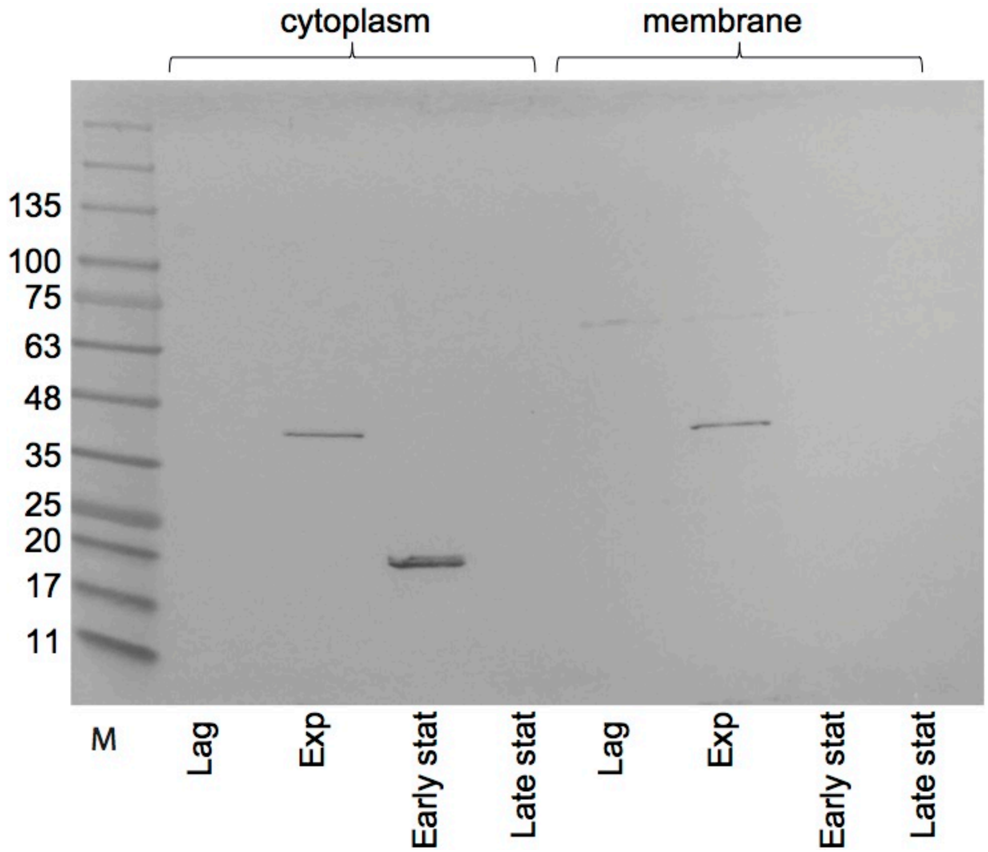

Figure S4. Detection of CwlM in cytoplasm and membrane fractions of *M. smegmatis* during different growth stages. Related to Figure 3. Bacteria were grown inoculated in 7H9 medium and incubated for 1 hour (lag-phase, Lag), grown to OD 0.8 (exponential phase, Exp), 3.6 (early stationary phase, Early stat) and 7.4 (late stationary phase, Late stat) at 37°C with shaking. Fractionation and Western blot analysis using the anti-CwlM antibody were done as described in Methods.

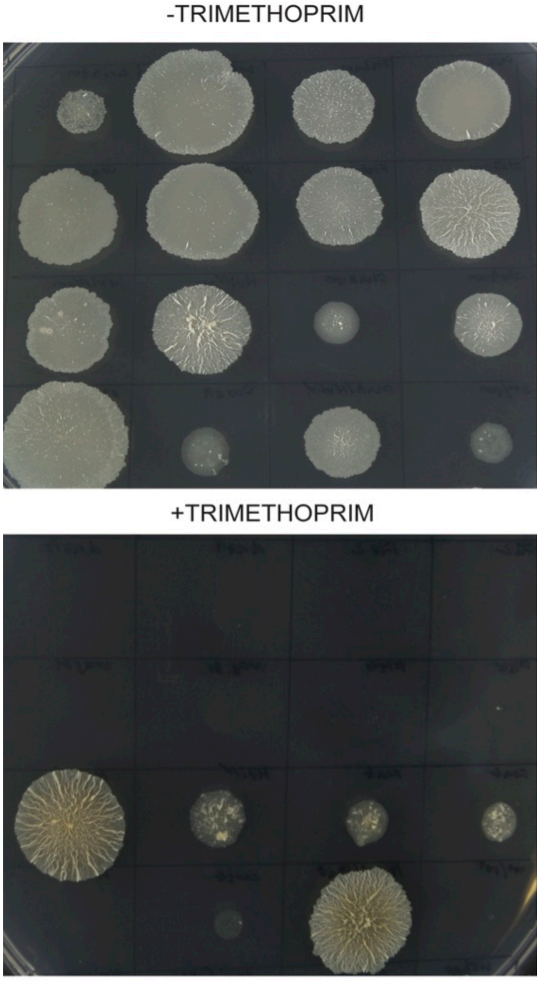

MAP

DnaA$_{400}$<br>CwlM$_{300}$	DnaA$_{200}$<br>CwlM$_{100}$	FtsE$_{400}$<br>CwlM$_{300}$	FtsE$_{200}$<br>CwlM$_{100}$
Wag31$_{400}$<br>CwlM$_{300}$	Wag31$_{200}$<br>CwlM$_{100}$	FtsZ$_{400}$<br>CwlM$_{300}$	FtsZ$_{200}$<br>CwlM$_{100}$
MurJ$_{icd400}$<br>CwlM$_{300}$	MurJ$_{icd200}$<br>CwlM$_{100}$	FhaA$_{400}$<br>CwlM$_{300}$	FhaA$_{200}$<br>CwlM$_{100}$
pUAB200<br>CwlM$_{100}$	CwsA$_{400}$<br>CwlM$_{300}$	MurJ$_{icd100}$<br>FhaA$_{200}$	pUAB300<br>pUAB400

Figure S5. Detection interactions of CwlM with other proteins by a mycobacterial protein fragment complementation assay. Related to Figure 5. See also Table S4. 300/400 is a negative empty plasmid control (pUAB300+pUAB400); CwlM/400 – control (pUAB300::*cwlM*+pUAB400); MurJ$_{ICD}$/FhaA – positive control (pUAB100:: *murJN$_{icd}$* +pUAB200::*fhaA*). Growth in the presence of trimethoprim indicates interactions between proteins fused to dihydrofolate-reductase domains. MAP shows the position of strains used in the assays (strains are detailed in Table S2).

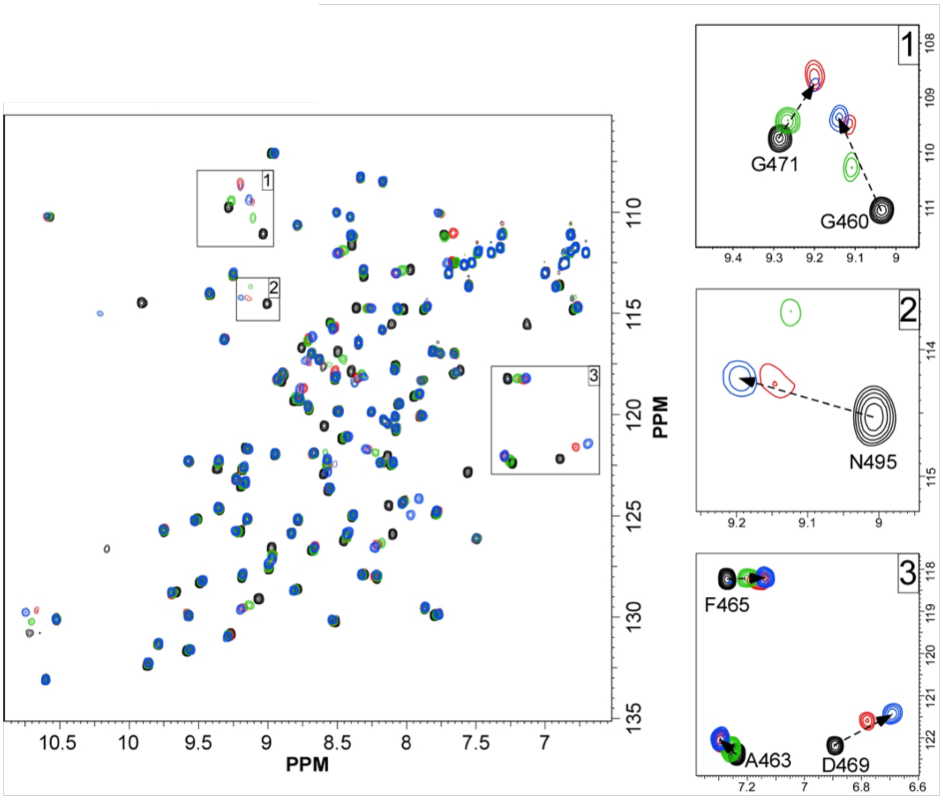

Figure S6. Superimposition of [^{1}H-^{15}N] HSQC spectra of Rv0020c FHA domain alone and in presence of 3 different phospho-peptides. Related to Figure 5. Superimpositions of [^{1}H-^{15}N] HSQC spectra of uniformly labelled samples Rv0020c FHA domain (50 μM) alone (black) and in presence of 50 μM of T382 phospho-peptide (red) or T386 phospho-peptide (green) or the double T382 and T386 phospho-peptide (blue). Three areas of the HSQC spectrum are expanded to the right. In each zoom, peaks are assigned and an arrow indicates the displacement of the peak when titrating with the double T382 and T386 phospho-peptide. The displacement observed for the double phosphor-peptide corresponds to the displacement observed for the T382 phospho-peptide.

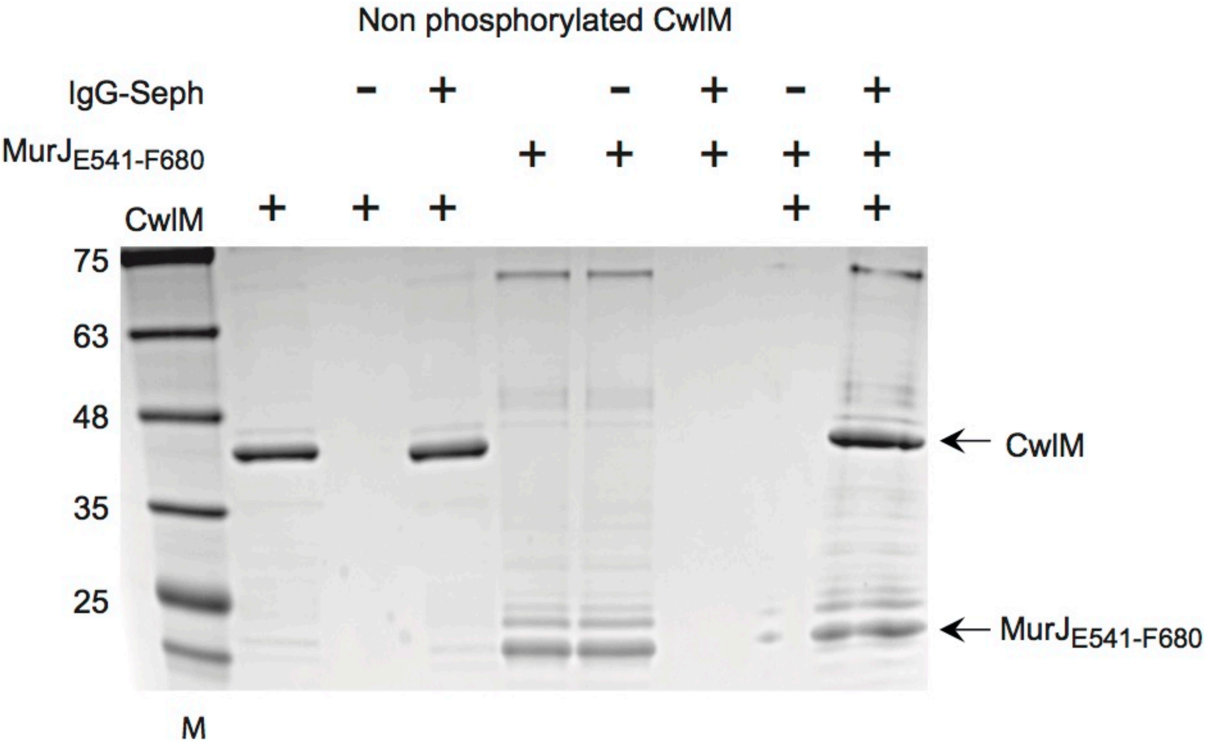

Figure S7. CwlM interacts with an E541-F680 linker of $MurJ_{ICD}$. Related to Figure 4. Recombinant proteins were immunoprecipitated and analysed by SDS-PAGE. M- protein markers; "+" – reagent added; "-" designates flow-through fractions.

SUPPLEMENTAL TABLES

Table S2. Strains generated in the study. Related to Figure 1-4.

Strain name	Strain description	Plasmid	Comments
***Mycobacterium tuberculosis* H37Rv strains**			
pknB-CM	PknB conditional mutant	pAZI9479::*pkn*	PknB depletion
cwlM-CM	CwlM conditional mutant	pAZI9479::*cwlM*	CwlM depletion
cwlM-CM$_{pMV}$	Empty plasmid control	pMV306	*cwlM*-CM complementation
cwlM-CM$_{WT}$	CM-CwlM wild type *cwlM* complemented	pMV306::*cwlM*	*cwlM*-CM complementation
cwlM-CM$_{T42A}$	T42A CwlM variant	pMV306::*cwlMT42A*	*cwlM*-CM complementation
cwlM-CM$_{T43A}$	T43A CwlM variant	pMV306::*cwlMT43A*	*cwlM*-CM complementation
cwlM-CM $_{T42A+T43A}$	T42A+T43A CwlM variant	pMV306::*cwlMT42A +T43A*	*cwlM*-CM complementation
cwlM-CM $_{T94A}$	T94A CwlM variant	pMV306::*cwlMT94A*	*cwlM*-CM complementation
cwlM-CM $_{T382A}$	T382A CwlM variant	pMV306::*cwlMT382 A*	*cwlM*-CM complementation
cwlM-CM $_{T384A}$	T384A CwlM variant	pMV306::*cwlMT384 A*	*cwlM*-CM complementation
cwlM-CM $_{T386A}$	T386A CwlM variant	pMV306::*cwlMT386 A*	*cwlM*-CM complementation
cwlM-CM $_{T382A+T384A}$	T382A +T384A CwlM variant	pMV306::*cwlMT382 A+384A*	*cwlM*-CM complementation
cwlM-CM $_{T382A+T384A+T386A}$	T382A +T384A+T386A CwlM variant	pMV306::*cwlMT382 A+384A+T386A*	*cwlM*-CM complementation
cwlM-CM $_{T43A+T382A+T386A}$	T43A+T382A +T384A CwlM variant	pMV306::*cwlMT43A +T382A+386A*	*cwlM*-CM complementation
cwlM-CM $_{T384A+T386A}$	T384A +T386A CwlM variant	pMV306::*cwlMT384 A+T386A*	*cwlM*-CM complementation
cwlM-CM$_{T43D}$	T43D CwlM variant	pMV306::*cwlMT43D*	*cwlM*-CM complementation
cwlM-CM$_{T382D}$	T382D CwlM variant	pMV306::*cwlMT382 D*	*cwlM*-CM complementation
cwlM-CM$_{T386D}$	T386D CwlM variant	pMV306::*cwlMT386 D*	*cwlM*-CM complementation
cwlM-CM $_{T382D+T386D}$	T382D+T386D CwlM variant	pMV306::*cwlMT382 D+T386D*	*cwlM*-CM complementation
cwlM-CM $_{T43D+T382D +T386D}$	T43D+T382D+T386D CwlM variant	pMV306::*cwlMT43D +T382D+T386D*	*cwlM*-CM complementation
cwlM-CM$_{R2}$	Truncated CwlM version (M1- K370)	pMV306::*cwlMR2*	*cwlM*-CM complementation
***Mycobacterium smegmatis* mc^{2}155 strains**			
pUAB300+ pUAB400	Empty plasmid control	pUAB300 pUAB400	Mycobacterial protein fragment complementation (M-PFC)

$CwlM_{100}$/ $DnaA_{200}$	Interaction strain	pUAB100::*cwl* pUAB200::*dnaA*	M-PFC
$CwlM_{300}$/ $DnaA_{400}$	Interaction strain	pUAB300::*cwlM* pUAB400::*dnaA*	M-PFC
$CwlM_{100}$/ $FtsE_{200}$	Interaction strain	pUAB100::*cwlM* pUAB200::*ftsEA*	M-PFC
$CwlM_{300}$/ $FtsE_{400}$	Interaction strain	pUAB300::*cwlM* pUAB400::*ftsE*	M-PFC
$CwlM_{100}$/ $FtsZ_{200}$	Interaction strain	pUAB100::*cwlM* pUAB200::*ftsZ*	M-PFC
$CwlM_{300}$/ $FtsZ_{400}$	Interaction strain	pUAB300::*cwlM* pUAB400::*ftsZ*	M-PFC
$CwlM_{100}$/ $Wag31_{200}$	Interaction strain	pUAB100::*cwlM* pUAB200::*wag31*	M-PFC
$CwlM_{300}$/ $Wag31_{400}$	Interaction strain	pUAB300::*cwlM* pUAB400::*wag31*	M-PFC
$CwlM_{100}$/ $FhaA_{200}$	Interaction strain	pUAB100::*cwlM* pUAB200::*fhaA*	M-PFC
$CwlM_{300}$/ $FhaA_{400}$	Interaction strain	pUAB300::*cwlM* pUAB400::*fhaA*	M-PFC
$CwlM_{100}$/ $MurJ_{icd200}$	Interaction strain	pUAB100::*cwlM* pUAB200::*$murJ_{icd}$*	M-PFC
$CwlM_{300}$/ $MurJ_{icd400}$	Interaction strain	pUAB300::*cwlM* pUAB400::*$murJ_{icd}$*	M-PFC
$CwlM_{300}$/ $CwsA_{400}$	Interaction strain	pUAB300::*cwlM* pUAB400::*cwsA*	M-PFC
$MurJ_{icd100}$/ $FhaA_{200}$	Interaction strain	pUAB100:: *$murJ_{icd}$* pUAB200::*fhaA*	M-PFC
$CwlM_{100}$/200	Interaction strain	pUAB100::*cwlM* pUAB200	M-PFC
$CwlM_{300}$/ 400	Interaction strain	pUAB300::*cwlM* pUAB400	M-PFC
$MurJ_{icd200}$/ 100	Interaction strain	pUAB100 pUAB200::*$murJ_{icd}$*	M-PFC
$MurJ_{icd400}$/300	Interaction strain	pUAB300 pUAB400::*$murJ_{icd}$*	M-PFC
C41(DE3) pET CwlM	6xHis-CwlM expression strain	pET15bTEV::*cwlM*	M-PFC
C41 (DE3) pET $MurJ_{icd}$	6xHis-$MurJ_{icd}$ expression strain	pET15bTEV::*$murJ_{icd}$*	Recombinant $MurJ_{icd}$
C41 (DE3)pET $MurJ_{E541-F680}$	6xHis-$MurJ_{E541-F680}$ expression strain	pET15bTEV::*murJ* *E541-F680*	Recombinant $MurJ_{E541-F680}$
C41 (DE3) pGEX Wag31	GST-Wag31 expression strain	pGEX::*wag31*	Recombinant Wag31
C41 (DE3) pGEX FhaA	GST-FhaA expression strain	pGEX::*fhaA*	Recombinant FhaA
BL21 (DE3) pGEX PknB	GST-PknB kinase domain expression strain	pGEX::*pknB kinase*	Recombinant PknB kinase domain

Table S3. Effect of threonine mutations on growth of *M. tuberculosis* in various media.

Related to Figures 2 and S3.

Strain	HK 7H10 agar	HKP 7H10 agar	HK 7H9 liquid	HKP 7H9 liquid	SMM	SMP
CM-CwlM$_{pMV}$	-	+++	-	+++	-	++
CM-CwlM$_{WT}$	+++	+++	+++	+++	+++	+++
CM-CwlM$_{T43A}$	+++	+++	+++	+++	+++	+++
CM-CwlM$_{T382A}$	-	+++	-	+++	++	+++
CM-CwlM$_{T386A}$	++	+++	++	+++	+++	
CM-CwlM$_{T382A+T386A}$	No colonies	No colonies	N/A	N/A	N/A	N/A
CM-CwlM$_{T382A+T386A+T43A}$	-	+++	-	+++	++	+++
CM-CwlM$_{T42A}$	+++	+++	+++	+++	+++	+++
CM-CwlM$_{T42A+T43A}$	+++	+++	++	+++	+++	+++
CM-CwlM$_{T94A}$	+++	+++	+++	+++	+++	+++
CM-CwlM$_{T384A}$	+++	+++	+++	+++	+++	+++
CM-CwlM$_{T382A+T384A}$	-	+++	-	+++	++	+++
CM-CwlM$_{T384A+T386A}$	+++	+++	+++	+++	+++	+++
CM-CwlM $_{T382A+T384A+T386A}$	-	Small colonies	N/A	N/A	N/A	N/A
CM-CwlM$_{T43D}$	+++	+++	+++	+++	+++	+++
CM-CwlM$_{T382D}$	++	+++	++	+++	++	+++
CM-CwlM$_{T386D}$	+++	+++	+++	+++	+++	+++
CM-CwlM$_{T382D+T386D}$	-	+++	-	+++	-	+++
CM-CwlM$_{T382D+T386D+T43D}$	-	+++	-	+++	++	-
CM-CwlM$_{T42D+T43D}$	+++	+++	+++	+++	+++	+++
CM-CwlM$_{T382D+T384D}$	-	+++	-	+++	N/D	N/D
CM-CwlM$_{R2}$	-	++	-	++	-	++

N/A – not applicable; N/D – not determined; “+++” - good growth compared with WT Mtb strain; “++” or “+” growth with defect; - no growth; “HK” with addition of hygromycin and kanamycin; “HKP” with addition of hygromycin, kanamycin and pristinamycin; SMM – sucrose magnesium medium.

Table S4. Identification of potential CwlM partners by immuno-precipitation and protein fragment complementation assays. Related to Figure 4.

Protein	Gene	Function	Fraction	M-PFC
DnaA	*Rv0001*	Chromosomal replication initiator protein	Cytoplasm	N
CwsA	*Rv0008c*	Membrane protein involved in division	Membrane	Y
FhaA	*Rv0020c*	Conserved protein with FhaA	Cytoplasm	Y
MurA	*Rv1315*	UDP-N-acetylglucosamine 1-carboxyvinyltransferase	Cytoplasm	Not assessed
Wag31	*Rv2145c*	DivIVa family protein	Cytoplasm	N
FtsZ	*Rv2150c*	Cell division protein	Cytoplasm	N
FtsE	*Rv3102c*	Putative cell division ATP-binding protein	Membrane	N
MviN	*Rv3910*	Potential Lipid II flippase MurJ	Membrane	Y

N – no growth on trimethoprim plates; Y- interaction.

Table S5. Primers used in the study. Related to Figures 2-4, S2-S5.

PRIMER	SEQUENCE 5'-3'	DESCRIPTION
CMRv3915F	ACTGCCATGGGCCCGAGTCCGCGCGAA	*cwlM*-CM generation
CMRv3915R	ACGTGCATGCTCATGCGTCGGACGGACTACG	*cwlM*-CM generation
FG3106	CGTTGGCGGTGGCGGCAC	*cwlM*-CM confirmation
FG2224	CCGTACACCGTACAAGGAG	*cwlM*-CM confirmation
Rv3915pEF	ACACATATGCCGAGTCCGCGCCGCGAAGA	Recombinant CwlM
RV3915PETR1	ACAGGATCCTTAAGAACCGCCGAGTCTACCCG	Recombinant CwlM
3915PMV306F2	ACTGGTACC AGCCGGTGAAACGAATCGTT	*cwlM*-CM complementation
3915PMV306R1	TACAAGCTTTAAGAACCGCCGAGTCTACC	*cwlM*-CM complementation
3915PMV306R3	TACAAGCTTTCAAACGGCTGTATCTGTTA	*cwlM*-CM complementation
3915T43AF	CAGGAAGAAGACCTGACGGCGGGCCGTAACGTCGCCCTT	CwlM SDM
3915T43AR	AAGGGCGACGTTACGGCCCGCCGTCAGGTCTTCTTCCTG	CwlM SDM
3915T43DF	CAGGAAGAAGACCTGACGGATGGCCGTAACGTCGCCCTT	CwlM SDM
3915T43DR	AAGGGCGACGTTACGGCCATCCGTCAGGTCTTCTTCCTG	CwlM SDM
3915T382AF	GCAAGAACGATCGGCCCGCTGGCACATTCACTTTCGC	CwlM SDM
3915T382AR	GCGAAAGTGAATGTGCCAGCGGGCCGATCGTTCTTGC	CwlM SDM
3915T382DF	GGCAAGAACGATCGGCCCGACGGCACATTCACTTTCGCC	CwlM SDM
3915T382DR	GGCGAAAGTGAATGTGCCGTCGGGCCGATCGTTCTTGCC	CwlM SDM
3915T386AF	CGGCCCACCGGCACATTCGCTTTCGCCGAGTTGCTGGCC	CwlM SDM
3915T386AR	GGCCAGCAACTCGGCGAAAGCGAATGTGCCGGTGGGCCG	CwlM SDM
3915T386DF	CGGCCCACCGGCACATTCGATTTCGCCGAGTTGCTGGCC	CwlM SDM
3915T386DR	GGCCAGCAACTCGGCGAAATCGAATGTGCCGGTGGGCCG	CwlM SDM
3915T382-6AF	GCAAGAACGATCGGCCCGCTGGCACATTCGCTTTCG	CwlM SDM
3915T382-6AR	GCGAAAGCGAATGTGCCAGCGGGCCGATCGTTCTTGC	CwlM SDM
3915T382-6DF	CGGCCCGACGGCACATTCGATTTCGCCGAGTTGCTGGCC	CwlM SDM
3915T382-6DR	GGCCAGCAACTCGGCGAAATCGAATGTGCCGTCGGGCCG	CwlM SDM
3915T384AF2	ACGATCGGCCCACCGGCGCATTCACTTTCGCCGAGTT	CwlM SDM
3915T384AR2	AACTCGGCGAAAGTGAATGCGCCGGTGGGCCGATCGT	CwlM SDM
2AtailF	AACGATCGGCCCGCTGGCGCATTCACTTTCGCCGAGTTG	CwlM SDM
2AtailR	CAACTCGGCGAAAGTGAATGCGCCAGCGGGCCGATCGTT	CwlM SDM
2AendF	ACGATCGGCCCACCGGCGCATTCGCTTTCGCCGAGTT	CwlM SDM
2AendR	AACTCGGCGAAAGCGAATGCGCCGGTGGGCCGATCGT	CwlM SDM
3AtailF	AACGATCGGCCCGCTGGCGCATTCGCTTTCGCCGAGTTG	CwlM SDM
3AtailR	CAACTCGGCGAAAGCGAATGCGCCAGCGGGCCGATCGTT	CwlM SDM
T42AF	CATCAGGAAGAAGACCTGGCGACGGGCCGTAACGTCGCC	CwlM SDM
T42AR	GGCGACGTTACGGCCCGTCGCCAGGTCTTCTTCCTGATG	CwlM SDM
T42-43AF	CATCAGGAAGAAGACCTGGCGGCGGGCCGTAACGTCGCC	CwlM SDM
T42-43AR	GGCGACGTTACGGCCCGCCGCCAGGTCTTCTTCCTGATG	CwlM SDM
T94AF	TACCGGCTCGGGGCCCGCGCGCTGTACCACCAATTCGGC	CwlM SDM
T94AR	GCCGAATTGGTGGTACAGCGCGCGGGCCCCGAGCCGGTA	CwlM SDM
2DtailF	AACGATCGGCCCGACGGCGACTTCACTTTCGCCGAGTTG	CwlM SDM
2DtailR	CAACTCGGCGAAAGTGAAGTCGCCGTCGGGCCGATCGTT	CwlM SDM
T42DF	CATCAGGAAGAAGACCTGGACACGGGCCGTAACGTCGCC	CwlM SDM
T42DR	GGCGACGTTACGGCCCGTGTCCAGGTCTTCTTCCTGATG	CwlM SDM
UAB1003915F	ATAGGATCCATGCCGAGTCCGCGCCGCGAA	M-PFC
UAB1003915R	CACATCGATAGAACCGCCGAGTCTACCCGC	M-PFC

UAB200Wag31F	ACACAATTGATGCCGCTTACACCTGCCGAC	M-PFC
UAB200Wag31R	ACATCGATGTTTTTGCCCCGGTTGAATTGA	M-PFC
UAB200FhaAF	GACACAATTGATGGGTAGCCAGAAAAGGCT	M-PFC
UAB200FhaAR	AACGTCGACGTGCATGCGGACGATGATCTC	M-PFC
UAB200FtsEF	ACACAATTGATGATCACCCTGGACCATGTC	M-PFC
UAB200FtsER	GAC ATCGAT GCGATCCATCCCGTAGACGCC	M-PFC
UAB200FtsZF	ACACAATTGATGACCCCCCCGCACAACTAC	M-PFC
UAB200FtsZR	GAC ATCGATGCGGCGCATGAAGGGCGGCAC	M-PFC
UAB200DnaAF	CACAATTGTTGACCGATGACCCCGGTTCA	M-PFC
UAB200DnaAR	GAC ATCGATGCGCTTGGAGCGCTGACGGAT	M-PFC
UAB400FhaAF	ACAGAATTCGATGGGTAGCCAGAAAAGGCT	M-PFC
UAB400-FhaAR	CACAAGCTTTCAGTGCATGCGGACGATGAT	M-PFC
UAB200 MviNF1	ACACAATTGGCCGTGCGAGCCCGAATCAGGA	M-PFC
UAB200MviNR1	AACATCGATGTTGCGACGGCGGCGGGTGTA	M-PFC
UAB400MviNR1	GACATCGATCTAGTTGCGACGGCGGCGGGT	M-PFC
UAB100MviNF2	CACGTTAACATG GCCGTGCGAGCCCGAATCA	M-PFC
pETMviNF	CACCATATGGCCGTGCGAGCCCGAATCAGGA	Recombinant $MurJ_{icd}$
pETMviNR	GAC GCT AGCCTAGTTGCGACGGCGGCGGGTA	Recombinant $MurJ_{icd}$
FhaALEICF	TACTTCCAATCCATGGGTAGCCAGAAAAGGCTGGT	Recombinant FhaA
FhaALEICR	TATCCACCTTTACTGTCAGTGCATGCGGACGATGATCT	Recombinant FhaA
Wag31LeicF	TACTTCCAATCCATGCCGCTTACACCTGCCGACGT	Recombinant Wag31
Wag31LeicR	TATCCACCTTTACTGTCACTAGTTTTTGCCCCGGTTGA	Recombinant Wag31
RTPknBF1	TCAGAACGGAATCATCCACCGTGA	qRT-PCR
RTPknBR1	GCGATGCCGAAATCCATCACCTTT	qRT-PCR
PMV306F	TGGTATCTTTATAGTCCTGTC	pMV306-specific primer
pMV306R2	TAGTTAACTACGTCGACATCGA	pMV306-specific primer
MviNpETR3	CATGGATCCCTAGAACGCAATCGGCCCACG	Recombinant $MurJ_{E541-F680}$
MviNpETF2	TATCATATGGAGGCGCGGGCGGCGCTGGAT	Recombinant $MurJ_{E541-F680}$ l
